# NEMO Family of Proteins as Polyubiquitin Receptors: Illustrating Non-Degradative Polyubiquitination’s Roles in Health and Disease

**DOI:** 10.3390/cells14040304

**Published:** 2025-02-18

**Authors:** Chuan-Jin Wu

**Affiliations:** Center for Cancer Research, National Cancer Institute, National Institutes of Health, Bethesda, MD 20892, USA; wuchu@mail.nih.gov

**Keywords:** NEMO, optineurin, ABIN, NF-κB, ubiquitin, inflammation, interferon, autophagy, immune response, immunodeficiency, neurodegenerative diseases

## Abstract

The IκB kinase (IKK) complex plays a central role in many signaling pathways that activate NF-κB, which turns on a battery of genes important for immune response, inflammation, and cancer development. Ubiquitination is one of the most prevalent post-translational modifications of proteins and is best known for targeting substrates for proteasomal degradation. The investigations of NF-κB signaling pathway primed the unveiling of the non-degradative roles of protein ubiquitination. The NF-κB-essential modulator (NEMO) is the IKK regulatory subunit that is essential for IKK activation by diverse intrinsic and extrinsic stimuli. The studies centered on NEMO as a polyubiquitin-binding protein have remarkably advanced understandings of how NEMO transmits signals to NF-κB activation and have laid a foundation for determining the molecular events demonstrating non-degradative ubiquitination as a major driving element in IKK activation. Furthermore, these studies have largely solved the enigma that IKK can be activated by diverse pathways that employ distinct sets of intermediaries in transmitting signals. NEMO and NEMO-related proteins that include optineurin, ABIN1, ABIN2, ABIN3, and CEP55, as non-degradative ubiquitin chain receptors, play a key role in sensing and transmitting ubiquitin signals embodied in different topologies of polyubiquitin chains for a variety of cellular processes and body responses. Studies of these multifaceted proteins in ubiquitin sensing have promoted understanding about the functions of non-degradative ubiquitination in intracellular signaling, protein trafficking, proteostasis, immune response, DNA damage response, and cell cycle control. In this review, I will also discuss how dysfunction in the NEMO family of protein-mediated non-degradative ubiquitin signaling is associated with various diseases, including immune disorders, neurodegenerative diseases, and cancer, and how microbial virulence factors target NEMO to induce pathogenesis or manipulate host response. A profound understanding of the molecular bases for non-degradative ubiquitin signaling will be valuable for developing tailored approaches for therapeutic purposes.

## 1. Introduction

**Figure 1:**
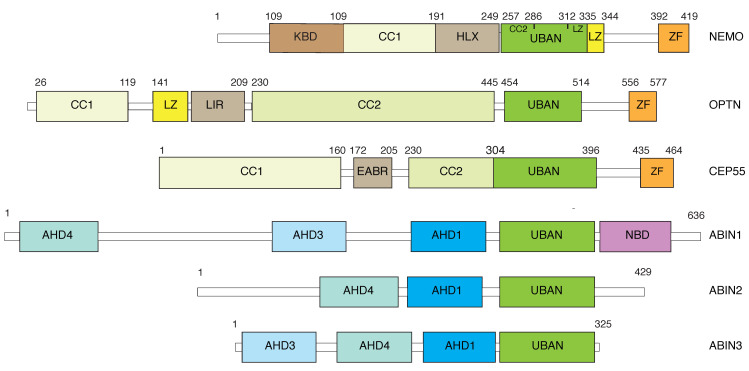
Schematic representation of the molecular structure and functional domains of the NEMO family of human proteins. The intervening domain (IVD, aa112–195) of NEMO mediates allosteric communication between the N- and C-terminal regions and conformational change that enhances IKKβ activation, following the interaction of NEMO with polyubiquitin. The N-terminal aa1–127 of optineurin is responsible for its interaction with TBK1. KBD: IKK-binding domain; HLX: helical domain; CC: coiled coil; LZ: leucine zipper; UBAN: ubiquitin binding in ABIN and NEMO; ZF: zinc finger; LIR: LC3 interaction region; EABR: ESCRT- and ALIX-binding region; AHD: ABIN homology domain; NBD: NEMO binding domain [8,9,10,18].

NEMO (NF-κB Essential Modulator, also known as IKKγ) is the regulatory subunit of the IκB kinase (IKK) complex and is essential for the canonical NF-κB pathway. The transcription factor NF-κB can be activated by a variety of exogenous and endogenous stimuli (infections, cytokines, stresses, etc.) and is involved in regulating a wide range of biological processes, including inflammation, immunity, and apoptosis [1]. NF-κB functions as a hetero- or homodimer of Rel family members, which include Rel-A (also known as p65), c-Rel, Rel-B, p50, and p52. In the unactivated state, NF-κB is sequestered in the cytoplasm by interaction with its inhibitory proteins, known as IκBs [2]. The canonical pathway leading to NF-κB activation requires the release of NF-κB from IκB, which is elicited by sequential post-translational modifications (PTMs) to IκB: phosphorylation by the two kinases, IKKα and IKKβ, which are together with NEMO to comprise the IKK complex, followed by ubiquitination and degradation in the proteasome [1]. The vast majority of the diverse stimuli utilize the canonical pathway to induce NF-κB activation, and it is on IKK that signaling for NF-κB activation converges [1,3,4,5]. Despite possessing no enzymatic activity, NEMO is essential for the activation of the two enzymatically active subunits of the IKK complex. How IKK is activated by upstream signaling was largely unknown until NEMO was discovered as a polyubiquitin chain receptor [6,7]. Yet, the finding helps solve an even larger puzzle, how signals emanating from diverse stimuli and receptors, which involve distinct sets of signaling proteins, result in the activation of this crucial intermediary. Shortly after the finding of NEMO–polyubiquitin interaction, other NEMO families of proteins, including optineurin, ABIN1, ABIN2, and ABIN3, were also revealed to have similar capacities of binding polyubiquitin chains [8,9]. Recently, the centrosomal protein 55 kDa (CEP55) was identified as another NEMO family-related protein that contains NEMO-like ubiquitin-binding domains [10] (Figure 1).

Ubiquitination is the process by which the evolutionarily conserved 76-amino-acid ubiquitin polypeptide is covalently attached to lysine residues in a target protein. This common form of post-translational modification is achieved by sequential catalyzation involving 2 ubiquitin-activating enzymes (E1s), ~40 ubiquitin-conjugating enzymes (E2s), and >600 ubiquitin ligases (E3s) in human cells, with ~100 deubiquitinating enzymes (DUBs) reversing the ubiquitin additions [11]. Protein ubiquitination is best known as a means of flagging substrates for proteasomal degradation, a pathway that is used in virtually every cellular process. Proteasome-mediated destruction requires the recognition of polyubiquitin chains formed by repeated conjugation of ubiquitin to itself at lysine (K)48 [12]. It also has long been known that proteins can be monoubiquitinated, and this modification plays a role in protein trafficking and histone modification [13]. Ubiquitin contains seven K residues (K6, K11, K27, K29, K33, K48, and K63), and all of them can serve as a site for linking with another ubiquitin, generating various isopeptide-linked ubiquitin chains [14]. The α-amino group on the N-terminal methionine 1 (M1) of ubiquitin can also be ubiquitinated, creating an additional chain type, M1-linked or linear ubiquitination [15]. The formation of polyubiquitin chains containing linkages other than K48 may lead to a variety of non-degenerative functional outcomes [15,16]. How these different patterns of ubiquitin modification acquire discrete functions depends largely on the specificity and function of ubiquitin receptors [17].

NEMO has constituted one of the most prominent receptors for non-degradative polyubiquitin chains. Due to being one of the earliest discovered non-degradative ubiquitin chain receptors, their versatility in sensing polyubiquitin chains of diverse linkages, the plethora of important roles of the NF-κB pathway, and many other functions in addition to modulating NF-κB activation, the NEMO family of proteins as ubiquitin receptors have been intensively studied. These studies have enormously advanced our knowledge of the functional roles and the underlying mechanisms of non-degradative polyubiquitination. Interestingly, NEMOs are frequent targets of protein components from various viruses. This review will provide an overview of how studies of the NEMO family of proteins as ubiquitin receptors have unfolded, with an emphasis on their promotion of understanding about non-proteolytic polyubiquitination in signaling cellular processes and its relevance to health and disease.

## 2. NEMO Is an Unconventional Type of Ubiquitin Receptor Possessing Chain Linkage Recognition Versatility

### 2.1. NEMO Binds Ubiquitin Chains

The human NEMO is a 419-amino-acid (aa) protein with several subdomains that are needed for NF-κB signaling (Figure 1). These domains include a primary N-terminal binding domain for IKKβ (KBD, aa 44–111), a ubiquitin-binding domain (UBAN, aa 257–335), and a C-terminal zinc finger domain (ZF, aa 392–419) [18,19,20] (Figure 1). NEMO oligomerizes and interacts with other proteins, such as IKK kinase subunits and IκB, via different domains, and its functional deficiencies in mediating NF-κB activation, which are caused by numerous mutations, are associated with inherited diseases, such as incontinentia pigmenti (IP), ectodermal dysplasia, and anhidrotic, with immunodeficiency (EDA-ID), and some cancers (robust details on NEMO’s domain composition, interaction proteins, and disease-associated mutations can be seen in the review article [21]).

In 2006, two studies reported that NEMO binds K63-linked polyubiquitin chains and, via mapping, identified a new ubiquitin-binding domain that was named UBAN, NUB, or CoZi [6,7]. In one case, efforts to identify binding partners for NEMO by a yeast two-hybrid screen led to the identification of cDNAs encoding 2–3 head-to-tail-linked ubiquitin molecules that come from typical ubiquitin mRNA transcripts. In vitro binding assays confirmed that NEMO binds linear di- but not mono-ubiquitin, and binds to synthesized K63-linked polyubiquitin chains [6]. The K63-linked polyubiquitin-binding activity of NEMO was also found in the course of studies aimed at identifying the ubiquitination sites of RIP1 to help understand the role of RIP1 ubiquitination in transmitting signaling to TNFα-induced NF-κB activation [7]. Notably, NEMO binds ubiquitin chains with K63-linkages far better than it binds K48-linked chains [6,7]. Yet, our study reported several additional features of NEMO–polyubiquitin interaction: a high affinity interaction of linear ubiquitin chains with NEMO, increased affinity with chain length, and association of defects in ubiquitin binding in NEMO mutation-caused diseases. These features suggest that NEMO may potentially interact with multiple types of polyubiquitin, especially under the condition that the ubiquitin chains are sufficiently long.

We used linear di-ubiquitin, whereas Ea et al. used K63-linked ubiquitin chains to map the NEMO ubiquitin-binding domain to a span that encompasses coiled-coil (CC) and leucine-zipper (LZ) domains and an intervening connecting peptide [6,7] (Figure 1). These studies also concluded that NEMO–polyubiquitin interaction is required for TNFα-induced IKK and NF-kB activation. Knowing that the linear ubiquitin chains have an apparently higher affinity than K63-linked ones, we were, however, short of a framework to conclude linear ubiquitination’s role in NF-κB signaling, as linear ubiquitin chains were regarded as non-physiological back then. Notably, linear ubiquitination was later found to be a real form of protein modification and plays an important role in IKK/NF-κB activation via sensing by NEMO (more detailed discussion below). This story constitutes an interesting reflection of the Hegel quote, “All that is real is reasonable, and all that is reasonable is real”. A later study revealed that NEMO contains another ubiquitin-binding site in the zinc-finger domain at the C-terminus [20]. In addition to linear and K63-linked polyubiquitin, NEMO also interacts with K11-linked ubiquitin chains [22], and K11-linked ubiquitinated RIP1 was reported a role in TNFα-induced NF-κB activation [23,24]. It appears that NEMO can also interact with K27-linked ubiquitin chains [25]. These, along with the report of NEMO interactions with hybrid ubiquitin chains (see below), exhibit the versatility of NEMO in interacting with polyubiquitin chains of other than K48-linkage.

### 2.2. Linear Ubiquitination and Hybrid Ubiquitination Are Natural Protein Modifications and Function in Mediating NF-κB Activation

For decades, linear ubiquitin chains were thought only to exist as the translation products of the polyubiquitin genes *UBB* and *UBC*, which, as precursors, are processed via cleavage by deubiquitinases into single ubiquitin molecules to provide the source for protein ubiquitination [26]. But in 2006, Kirisako et al. [27] identified a ligase complex that assembles ubiquitin chains in a head-to-tail or linear fashion through the N-terminal methionine (M1). This complex comprises HOIL-1, HOIP, and SHARPIN and was named the linear ubiquitin chain assembly complex (LUBAC). Three years later, LUBAC was determined to be involved in regulating the TNFα-activated canonical NF-κB pathway [28]. In addition to homotypic polyubiquitination, heterologous complex ubiquitination, with hybrid and branched chains, was also reported [29]. K63/M1-hybrid or K48/K63-branched ubiquitin chains regulate NF-κB signaling [30,31]. Additionally, non-Lys ubiquitination, such as thioester-linked ubiquitination of Cys residues and oxyester-linked ubiquitination of Ser/Thr residues, have been identified [32,33].

## 3. NEMO Family of Proteins Engage Ubiquitin Chains in Innate Immunity

The innate immune cells, such as dendritic cells, macrophages, and neutrophils, employ pattern-recognition receptors (PRRs) for the initial detection of microbes. PRRs recognize molecular signatures known as pathogen-associated molecular patterns (PAMPs) from microbes, as well as self-derived damage-associated molecules patterns (DAMPs) from damaged and necrotic cells. Several classes of PRRs have been identified, including Toll-like receptors (TLRs), RIG-I-like receptors (RLRs), Nod-like receptors (NLRs), AIM2-like receptors (ALRs), C-type lectin receptors (CLRs), and intracellular DNA sensors, such as cGAS [34,35]. PRRs activate downstream signaling pathways that enable innate immune responses by producing inflammatory cytokines, type I interferons (IFNs), and other mediators. These processes not only trigger immediate host defensive responses that help to clear infecting microbes but also instruct antigen-specific adaptive immune responses [36,37].

### 3.1. Toll-like Receptor Signaling-Induced NF-κB Activation

**Figure 2:**
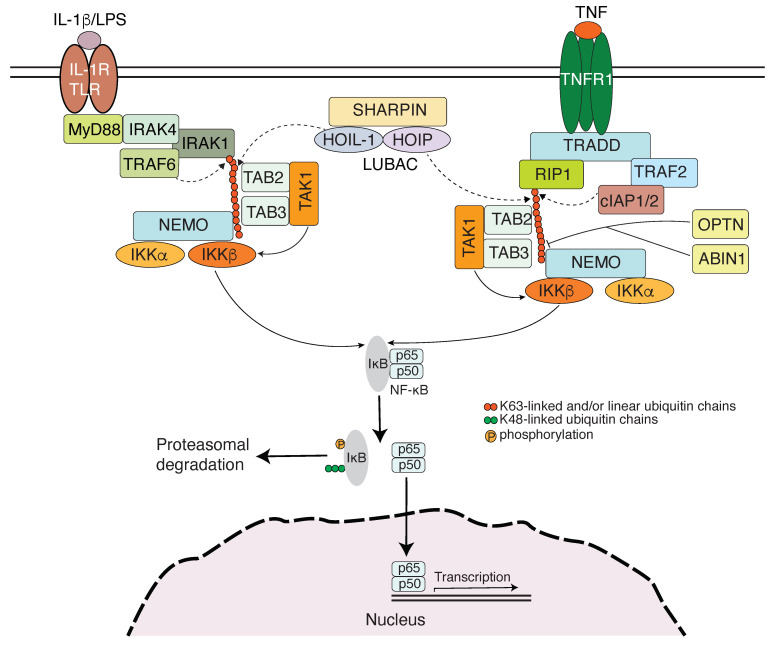
The signaling cascades generalized for ubiquitin-mediated IKK activation in IL-1R/TLR and TNF pathways. K63-linked ubiquitin chains modified to IRAK1 by TRAF6 in the IL-1R/TLR pathway or RIP1 by cIAP1/2 in the TNF pathway bind to TAB2/3 subunits of the TAK1 kinase complex, and this binding promotes autophosphorylation of TAK1, which results in its activation. In the meantime, the K63-linked and/or linear ubiquitin chains resulting from the LUBAC catalyzation on IRAK1 or RIP1 also bind to NEMO, bringing the IKK complex into the vicinity of TAK1, thereby facilitating the phosphorylation and subsequent activation of IKKβ by TAK1. An arrow with a curved dashed line indicates “ubiquitinate”, and an arrow with a curved solid line indicates “phosphorylate”. The different ubiquitin chain linkages are color-coded. The depiction of the signaling model is adapted from the articles [21,47].

TLRs recognize components such as lipopolysaccharides (LPS), DNA, and RNA from viruses, bacteria, and fungi to sense infections, and activate NF-κB and IRF transcription factors to trigger the transcription of genes coding for various cytokines and type I IFNs for immune response [38]. TLR signaling is dependent on MyD88, which is recruited to TLRs through TIR (Toll/interleukin-1 receptor) domain interaction. MyD88 binds to IRAK4, which recruits and phosphorylates IRAK1. IRAK1 then interacts with TRAF6, an E3 ubiquitin ligase. By cooperating with Ubc13 and Uev1A, a K63-specific E2 complex, TRAF6 generates K63-linked polyubiquitin chains, which have been shown to be attached to IRAK1, IRAK4, and MyD88 [30,39,40]. K63-polyubiquitin recruits the TAK1 kinase complex by binding to TAB2/3, and this recruitment promotes the autophosphorylation and activation of TAK1 [41]. K63-linked polyubiquitin also recruits the IKK complex by binding to NEMO, thereby promoting the phosphorylation of IKKβ by TAK1 [6,7,42]. In addition, LUBAC is also required for full activation of NF-κB by the MyD88-dependent pathway [43]. Linear polyubiquitin chains promote the activation of the IKK complex by interacting with NEMO [19,44] (Figure 2). It has been reported that LUBAC extends preexisting K63-linked polyubiquitin chains with M1-linked ones [30]. TLR3 signaling and part of TLR4 signaling, which are independent of MyD88 but via TRIF, also involve K63-linked and linear ubiquitin chains attached to signaling components that are recognized by NEMO in mediating NF-κB activation [45,46].

The physiological significance of polyubiquitin binding to ABIN1 is demonstrated by autoimmunity developed in the knock-in mice with the polyubiquitin binding-defective (D485N) mutant. The ABIN1 mutation-induced autoimmunity is suppressed by crossing with MyD88 knockout mice, suggesting that the suppression of TLR-MyD88 signaling by ABIN1 is needed for the phenotype to develop [48].

### 3.2. RLR-Mediated Interferon Production and Antiviral Response

The RLR family members recognize viral RNA and activate antiviral signaling pathways, leading to the production of type I IFNs and other pro-inflammatory cytokines [49]. The binding of viral RNA to RIG-I or MDA5 induces a dramatic conformational change that exposes the N-terminal CARD domains, which subsequentially interact with the CARD of the mitochondrial adapter protein MAVS (also named as ISP-1 or VISA) [50,51]. MAVS then aggregates and activates cytosolic protein kinases, including IKK and the IKK-like kinase TBK1, which activates NF-κB and IRF3, respectively, coordinately leading to type I IFN production [52]. Double-stranded RNA (dsRNA) formed during the replication of some RNA viruses may also interact with TLR3 to initiate signaling that is dependent on TRIF to lead to activation of TBK1 and IRF3 to trigger IFNβ gene transcription [53].

NEMO also has an important role in virus-induced type I IFN production. Children carrying hypomorphic mutations in NEMO are susceptible to the development of herpes simplex virus type 1 encephalitis owing to defects in the activation of IRF3 and the production of type I IFNs induced by TLR3 activation [54]. A study by Zhao et al. [55] showed that in the absence of NEMO, replication of the Sendai virus becomes rampant because of defective virus-induced IRF3 phosphorylation and type I IFN production. Reconstitution of NEMO-deficient cells with NEMO, but not with a polyubiquitin binding-defective mutant of NEMO, rescues the loss in IFNβ production induced by RIG-1 activation via Sendai virus infection [55]. Similarly, NEMO, but not a polyubiquitin binding-defective mutant, restores IRF3 phosphorylation and TBK1 activation in a reconstituted in vitro signaling network in which mitochondria derived from virus-infected cells activate IRF3 in cytosol preparation. Also, K63-linked polyubiquitination is required for the IRF3 activation by the viral infection in this in vitro system [56]. A later study deciphered that viral RNA-engaged RIG-I induces aggregation of MAVS on the mitochondrial membrane, and the MAVS aggregates are potent in activating IKK and TBK1 in the cytosol. Again, K63 polyubiquitination plays an important role in these processes [52].

One study reported that auto-ubiquitinated TRIM26 binds with NEMO to bridge TBK1-NEMO interaction, which leads to recruitment of TBK1 to the MAVS signalosome and activation of TBK1 [57]. Studies from other groups found that K63-linked ubiquitination of MAVS by TRIM31 promotes MAVS aggregation and recruitment and activation of IKK and TBK1 [58,59]. In addition, TRIM25 catalyzes the ubiquitination of MAVS, enabling recruitment of NEMO and TBK1 to mitochondria. MAVS degradation then releases NEMO and TBK1 into cytosol to activate IRF3 and type I IFN production [60]. TRIM25 is also required for K63-linked ubiquitination of RIG-1 and RIG-1 signaling-mediated antiviral activity [61]. Additionally, TRIM25 is involved in the signaling of MDA5 and its activation-mediated antiviral signaling. But it appears that MDA5 signaling involves TRIM25 via a different mechanism, with TRIM25 acting between MAVS and IKK and engaging TRAF6 in NF-κB activation [62]. LUBAC suppresses RIG-1-induced interferon production via downregulating TRIM25-mediated RIG-1 ubiquitination [63], whereas the E3 complex for linear ubiquitination is essential for MDA5-induced interferon production and regulates persistent murine norovirus infection [64]. It is also suggested that M1-ubiquitin chains of NEMO competed with MAVS to bind TRAF3, disrupting IRF3 signaling cascade and reducing IFN production [65].

Being in a complex with TBK1 and TRAF3, optineurin inhibits Sendai virus- and dsRNA- triggered induction of IFNβ and renders cells more susceptible to Semliki Forest virus (SFV) infection. Furthermore, the ubiquitin-binding activity is required for optineurin’s correct localization to the Golgi-associated compartment and its function in IFN production inhibition [66]. Conversely, multiple studies reported that optineurin recognizes seemingly K63-linked ubiquitinated TBK1 and is involved in positively regulating TBK1 activation and interferon production [67,68]. It has been proposed that at the Golgi apparatus, where part of optineurin is localized, ubiquitinated TBK1 is sensed by optineurin, causing TBK1 trans-autophosphorylation and activation after TLR3 or RLR stimulation [69]. The reason for the controversial roles of optineurin in interferon production is unknown. However, in vivo data from knockout models demonstrate that optineurin plays an important role in TBK1 activation and interferon production after TLR4 stimulation and restricts Salmonella infection [70,71].

The ubiquitin binding-defective ABIN1 (Q487H) mutant mice display hematopoietic deficiencies, manifested as anemia, thrombocytopenia, and megakaryocyte dysplasia, and co-deletion of the *Ifnar1* gene markedly ameliorates these exhibitions. This provides in vivo evidence indicating that ABIN1 inhibits type I IFN expression via ubiquitin binding [72].

### 3.3. Autophagy

Autophagy is a conserved pathway for the degradation of intracellular materials and the recycling of nutrients. The lysosomal degradation pathway of autophagy plays an essential role in cellular and tissue homeostasis by selectively removing aggregated proteins, dysfunctional organelles, and intracellular pathogens, and is thus considered as a component of innate immunity [73]. Optineurin has been identified as an autophagy receptor that connects the ubiquitinated autophagy substrates with LC3-positive phagophore membranes. Optineurin, as an autophagy receptor, was firstly shown to require its ubiquitin- and LC3- binding activity to target ubiquitin-coated cytosolic Salmonella enterica for autophagic clearance [74]. Studies show that optineurin is not only involved in the initiation but also in multiple steps of the autophagy process [75]. Optineurin is also involved in regulating xenophagy to restrict viruses, such as herpes simplex virus-1 (HSV-1), in which the infection plays a role in chronic neurodegeneration [76,77]. LUBAC-synthesized M1-linked polyubiquitin on cytosol-invading *Salmonella* Typhimurium recruits optineurin and NEMO, activating autophagy and NF-κB, respectively, to restrict the proliferation of the bacterium [78,79]. Optineurin is also required for xenophagy to restrict some mycobacteria, such as M. tuberculosis. Mechanistically, optineurin interacts with mycobacterial virulence factors and is colocalized with M. tuberculosis in macrophages [80]. A study using an in vivo zebrafish model showed that optineurin is required for autophagic host defense against Mycobacterium marinum infection that causes a tuberculosis-like disease in zebrafish [81]. Moreover, optineurin engages in ubiquitin binding to contribute to selective autophagy of depolarized mitochondria (mitophagy) [82,83], damaged lysosomes (lysophagy) [84], and protein aggregates (aggrephagy) [75,85]. The defects in clearing those damaged organelles or protein aggregates are associated with diseases, prominently, neurodegenerative diseases [83]. These autophagic processes require the recognition of K63-linked ubiquitinated and/or linear ubiquitinated cargo proteins by optineurin in collaboration with other ubiquitin receptors, such as CALCOCO2 and p62 [78,84,85].

NEMO, by engaging its ubiquitin-binding activity to act as an autophagy adapter, reshapes the α-Synuclein aggregate interface to favor its co-condensation with p62 and thus promotes the autophagosomal clearance of the α-Synuclein aggregates, whose accumulation causes neurological symptoms [86]. ABIN1 binds LC3B and promotes mitophagy [87], but whether this regulation involves ABIN1′s ubiquitin-binding activity has not been determined.

## 4. Ubiquitin Chains Engage NEMO in Adaptive Immunity

**Figure 3:**
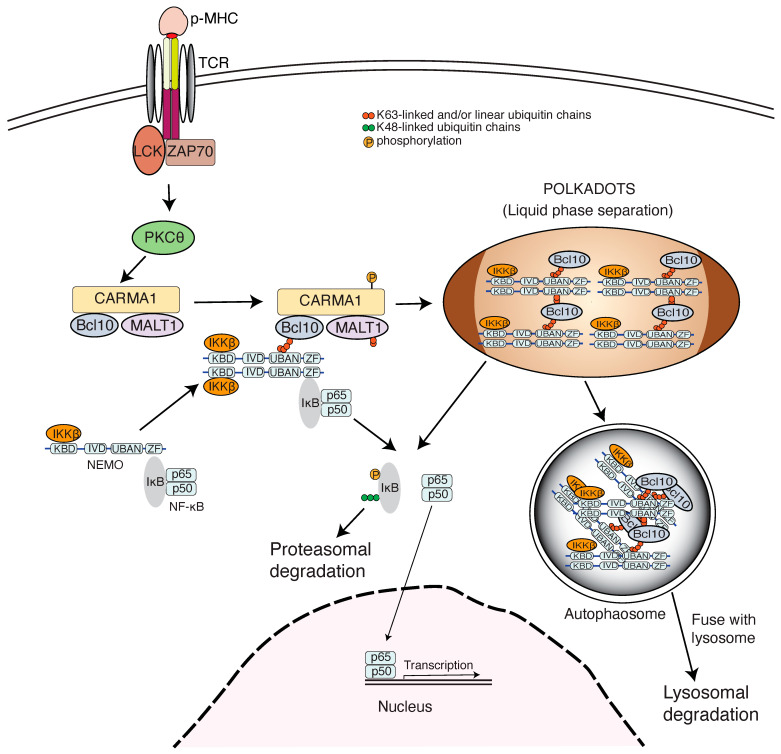
Ubiquitin-mediated IKK activation and Bcl10 autophagy in the T cell signaling pathway. The binding of the TCR to the antigen peptide presented by MHC molecules (p-MHC) induces T cell activation and leads to the initiation of a T cell response. When TCR:pMHC binding occurs, the TCR complex is phosphorylated by Lck. Phosphorylated signaling motifs in the TCR complex recruit the ZAP70 kinase. The recruitment of ZAP70 and its phosphorylation by Lck causes its activation. ZAP70 then phosphorylates the adaptor protein LAT, which recruits additional signaling effectors that become activated, leading to PKCθ activation. The activated PKCθ phosphorylates CARMA1 in the CARMA1-Bcl10-MALT1 complex, inducing K63-linked or linear ubiquitination of Bcl10, which in turn binds to NEMO in the IKK complex. The formation of supercomplexes leads to liquid phase separation, which is presented as punctate cytosolic structures called POLKADOTS under a microscope [104]. The liquid phase separation and ubiquitin-mediated NEMO conformational changes induce IKKβ activation that triggers IκB phosphorylation, ubiquitination, and proteasomal degradation, then NF-κB activation. Subsequently, the complexes encompassing ubiquitinated Bcl10 undergo autophagy that leads to Bcl10 lysosomal degradation and the inhibition of T cell signaling-induced NF-κB and MAPK activation.

A variety of murine genetic models have demonstrated that NF-κB plays important roles in many aspects of T cell development, activation, and effector functions [88]. TCR proximal signaling involves activation of the Src (Lck and Fyn) and Syk (ZAP70) families of protein tyrosine kinases that phosphorylate adaptor proteins, such as LAT and SLP76, leading to the activation of protein kinase C (PKC) θ [89,90]. PKCθ phosphorylates CARMA1, altering its conformation and promoting the assembly of the CARMA1-Bcl10-MALT1 (CBM) complex that, in turn, activates IKK [91]. Ubiquitin binding-defective mutants could not restore T cell activation-induced NF-κB activation in NEMO-deficient T cells [92,93], indicating that the ability of NEMO to bind ubiquitin is critical in the process. The polyubiquitination of the two components of the CBM complex, MALT1 and Bcl10, were reported to be essential for TCR activation-induced IKK/NF-κB activation. The study by Wu et al. demonstrated that Bcl10 ubiquitination occurs on K31 and K63, and ubiquitinated Bcl10 acts as the major component in CBM that interacts with NEMO to link IKK to CBM. Deficiency in Ubc13, which is a specialized E2 for catalyzing K63-linked ubiquitination, leads to defective T cell proliferation and impaired TCR-mediated activation of NF-κB, JNK, and p38 [94]. This suggests that K63-linked protein ubiquitination is involved in these TCR signaling pathways. The functional significance of K63-linked ubiquitination in T cells is highlighted by the marked reduction of peripheral T cells in T cell-conditional Ubc13 knockout mice [94]. The K63-linked Bcl10 ubiquitination could be mediated by the E3 ligases c-IAP1 and c-IAP2 [95]. The CARMA1–Bcl10 complex also mediates JNK2 activation in the TCR signaling pathway [96], and this is also dependent on Bcl10 ubiquitination [93]. Additionally, LUBAC was found to be in a complex with the CBM complex and regulates antigen receptor-induced NF-κB activation, but in a catalytic activity-independent fashion [97], whereas others reported that the LUBAC-mediated linear ubiquitination of Bcl10 is required for IKK activation by TCR signaling and oncogenic CARMA1 [98] (Figure 3).

The regulatory T cell (Treg)-specific knockout of Ubc13 impairs the suppressive function of Treg cells and leads to the development of autoimmune symptoms in mice [99], indicating that K63-linked ubiquitination plays a vital role in regulating Treg cells. This regulation involves IKK activation since expression of a constitutively active form of IKKβ rescues the function of Ubc13-deficient Treg cells [99]. LUBAC has also been reported roles in regulating Treg. A study revealed that SHARPIN is required for Treg development [100], and another one showed that the LUBAC components HOIP, HOIL-1, and SHARPIN have essential roles in late thymocyte differentiation, Treg development, and Treg homeostasis [101]. In line with this, the deubiquitinase CYLD, which hydrolyzes K63-linked and linear ubiquitin chains to downregulate IKK/NF-κB action, inhibits Treg development [102].

Optineurin is transiently lost in response to TCR stimulation. Overexpression and siRNA knockdown experiments show that optineurin down-regulates TCR-induced NF-κB activation and TNFα production in a manner dependent on ubiquitin-binding [103].

## 5. Ubiquitin Chains Engage NEMO Family of Proteins in Inflammation

NF-κB is a master transcription factor in modulating inflammatory response throughout the body [105]. TNFα is a pro-inflammatory cytokine with multiple roles in innate and adaptive immune responses, and biologics that target TNF and TNF receptors are among the most successful drugs for the treatment of chronic inflammatory and autoimmune diseases [106,107]. NF-κB activation is responsible for much of the TNF-induced inflammatory response, and the TNF receptor 1 (TNFR1)-signaling pathway is the most well-studied canonical NF-κB pathway. TNFR1 occupancy in the presence of TNFα results in its trimerization and the serial recruitment of the TNF receptor-associated death domain (TRADD), receptor interacting protein (RIP) 1, and TNFR-associated factors (TRAFs)-2 and 5 and cIAP-1 and cIAP-2 [108]. cIAPs, as E3 ligases, generate K63-linked polyubiquitin chains on RIP1 [109,110]. The K63-linked polyubiquitin chains then recruit the TAK1-TAB complex and the IKK complex to the receptor complexes via binding to TAB2/3 [41] or NEMO [6,7], respectively. In the meantime, LUBAC is also recruited to the TNFR1 and generates M1-linked polyubiquitin on RIP1 and NEMO [28,111,112,113]. These M1-linked polyubiquitin chains also function as a scaffold to recruit the IKK complex via the ubiquitin-binding domain of NEMO [6,19,44]. Within the TNFR1 signalosome complex, TAK1 activates the IKK by phosphorylation [114] (Figure 2). The reconstitution of NEMO-deficient cells with wild-type or ubiquitin-binding defective mutants of NEMO indeed demonstrated that the NEMO–ubiquitin interaction is important for TNFα-induced IKK/NF-κB activation [6,7]. The finding that NEMO binds linear and K63-linked polyubiquitin provided the possible missing link between TNFR1 occupancy and IKK activation. IL-1 is also a key regulator of inflammation via activating NF-κB and modulating a variety of innate immune processes [115]. The IL-1 receptor has a TIR domain, which also exists in TLRs and is responsible for MyD88 recruitment, thereby sharing with TLR signaling in activating NF-κB [39]. As discussed above, NEMO, as a ubiquitin receptor, plays a critical role in TLR/IL-1R-mediated NF-κB activation (Figure 2).

LUBAC is required for the assembly of NLRP3/ASC inflammasome, and ASC is identified as the LUBAC substrate in the regulation of inflammasome activation. The underlying mechanism is unknown, however, the ubiquitinated ASC is capable of binding to NEMO [116]. Mitochondrial outer membrane permeabilization (MOMP) has been shown to be pro-inflammatory and may trigger anti-tumor immunity. Mechanistically, upon MOMP, many proteins localized to either inner or outer mitochondrial membranes are ubiquitinated and then recruit NEMO to activate NF-κB [117].

Optineurin has been shown in vitro to downregulate TNF signaling-mediated NF-κB activation by competing with NEMOs for polyubiquitin [8]. However, this regulation has not been verified by in vivo data [70]. An in vitro overexpression experiment showed that ABIN-1 inhibits TNFα- and IL-1-induced NF-κB activation [118]. This role was also confirmed by a murine asthma model, in which the adenoviral gene transfer of ABIN1 decreased allergic airway inflammation [119]. The inhibition of NF-κB action by ABINs is mediated by their ubiquitin binding [9]. ABIN-1 provides a critical link between M1 ubiquitination mediated by the LUBAC complex and K63 deubiquitination by phospho-A20 to modulate the activation of RIP1 [120]. A recent study reveals that optineurin-mediated selective autophagy targets NLRP3 for degradation via binding to K27-linked polyubiquitin chains on the protein. This process diminishes NLRP3 inflammasome and may be harnessed to alleviate inflammatory bowel disease [121]. Taken together, ample evidence indicates that the ubiquitin-binding activities of the NEMO family of proteins critically regulate inflammatory processes and may be a viable target for clinical benefit.

## 6. Ubiquitin Chains Engage NEMO in DNA Damage Response

Genotoxic stress induced by chemotherapy or radiation therapy triggers the ATM-dependent translocation of NEMO from the nucleus to the cytosol and subsequent IKK/NF-κB activation. In multiple tumor types, activation of the transcription factor NF-κB increases the resistance of tumor cells to anticancer therapies and contributes to tumor progression [122]. DNA damage stimulates the formation of a cytosolic complex containing ATM, NEMO, RIP1, and TAK1. ATM-induced TAK1 activation is dependent on NEMO and SUMO-1 and ubiquitin modification of RIP1 [122]. Another study reported that Bcl10 recruits Ubc13 to enhance polyubiquitination of RNF8/RNF168 and mediates double strand break (DSB) signaling and repair [123]. LUBAC-dependent NEMO linear ubiquitination is also involved in DNA damage-initiated NF-κB activation and protects cells from DNA damage-induced apoptosis [124].

## 7. Ubiquitin Chains Engage NEMO in Cancer

The NF-κB pathway plays a critical role in cancer. NF-κB activity promotes tumor cell proliferation, suppresses apoptosis, and attracts angiogenesis, but it also induces epithelial–mesenchymal transition to facilitate metastasis [125]. A piece of convincing evidence supporting NF-κB’s role in tumor development is that loss-of-function mutations in CYLD, which is known for its deubiquitinase activity in inhibiting NF-κB signaling, leads to spontaneous development of skin cancers. Recognized as a tumor suppressor, dysregulation in CYLD is also associated with many other types of cancers [126]. Likewise, A20 is known to function as a tumor suppressor in several lymphomas, including marginal zone lymphoma, diffuse large B cell lymphoma, and mucosa-associated lymphoid tissue (MALT) lymphoma, by acting as a deubiquitinase that inhibits NF-κB activation [127]. More than 70% of Epstein–Barr virus (EBV)-negative Hodgkin lymphoma cases display inactivation of the deubiquitinase A20, involving linear ubiquitin chain assembly by LUBAC in the oncogenesis [128].

Oncogenic CARMA1 variants linked with diffuse large B cell lymphoma spontaneously induce linear ubiquitination of Bcl10 that correlates with their abilities to activate NF-κB [98]. Human T-cell leukemia virus type 1 (HTLV-1) is a complex retrovirus that infects CD4+ T cells and causes adult T-cell leukemia/lymphoma, largely by its Tax protein-triggered NF-κB activation [129]. Tax interacts with Ubc13 and is conjugated on C-terminal lysine residues with lysine 63-linked polyubiquitin chains. K63-linked polyubiquitin chains on Tax may serve as a platform for signaling complexes since this modification is critical for Tax interaction with NEMO and IKK activation [130]. The lipid raft protein FLOT1 is upregulated in esophageal squamous cell carcinoma (ESCC) samples from patients, and it has been shown that FLOT1 promotes ESCC cell proliferation and tumor growth in mice. FLOT1 activates TNFα receptor signaling and sustains activation of NF-κB in ESCC cells by inducing K63-linked ubiquitination of signaling intermediaries in the TNF signaling pathway [131]. The ubiquitin-binding proteins Epsin-1 and Epsin-2 are upregulated in breast cancers. They interact with LUBAC and promote NEMO linear ubiquitination, leading to the heightened IKK activation and sustained NF-κB signaling that promotes breast cancer development [132]. Golgi phosphoprotein 3 (GOLPH3) is frequently upregulated in hepatocellular carcinoma (HCC). It promotes hepatocellular carcinoma cell aggressiveness by enhancing NF-κB activation via the induction of K63-linked ubiquitination of TRAF2, RIP1, and NEMO [133]. As such, oncogenes and microbial virulence proteins may harness NEMO’s ubiquitin-binding activities to induce diverse types of cancers.

## 8. The Mechanisms for Non-Degradative Ubiquitination in Mediating IKK Activation

The diverse stimuli that use the canonical pathway utilize distinct sets of proteins to propagate signals in the same IKK activation, then NF-κB activation [1]. It has become clear that the recognition of polyubiquitinated proteins by NEMO is a conserved mechanism for the signaling pathways that use the canonical way to activate NF-κB. Several evolving mechanisms have been proposed for the NEMO–polyubiquitin interaction in mediating IKK activation. The NEMO binding targets identified so far are either not kinases or, like RIP1 and IRAK1, are kinases, but their kinase activities are dispensable for IKK activation [134,135]. The ubiquitinated proteins may act as adaptors to bring the IKKKs into the proximity of IKK. Among the IKKKs, TAK1 is obviously a candidate, especially after gene targeting experiments confirmed that TAK1 is required for TNF- and IL-1-induced NF-κB activation [136]. Unanchored K63 polyubiquitin chains were found to directly activate the TAK1 in vitro through binding to the TAB2 or TAB3. The activated TAK1 then phosphorylates IKKβ and leads to IKK activation [137]. The phosphorylation of IKKβ by TAK1 requires NEMO and TAB2, as well as unanchored polyubiquitin chains [138]. It has been shown that TAB2/TAB3 only interact with K63-linked but not linear ubiquitin chains [139,140,141]. Therefore, an attractive model is that TAB2/TAB3, in the TAK1 complex, bind to K63-linked ubiquitin chains, and NEMO in the IKK complex binds to linear or K63-linked chains of the same ubiquitinated targets, such as RIP1, to bring TAK1 and IKK into proximity so that TAK1 phosphorylates IKK and activates IKK [7,41] (Figure 2).

However, some questions regarding this model remain to be clarified. Although the siRNA knockdown of both TAB2 and TAB3 shows a defect in NF-κB activation [41], knockout of TAB2 has no effect on TNFα-stimulated NF-κB activation [136]. Yet, the TAB2 and TAB3 double knockout B cells and macrophages have normal NF-κB, and even TAK1 activation, in response to TLR stimulation [142]. More uncertain is that TAB2/TAB3, and even TAK1, are universal requirements, like NEMO, for all the canonical NF-κB pathways. Despite the existence of conflicting observations, there are reports suggesting that TAK1 is dispensable for TCR-dependent NF-κB activation in effector T cells [143] or BCR-induced NF-κB activation [144] in TAK1 conditional knockout mice. Notably, a constitutively active form of IKKβ ceases to activate NF-κB in cells in the absence of NEMO [145]. This model has a weakness in explaining the cases where only linear ubiquitination is involved, especially in a situation where the ubiquitinated protein is exogenous, e.g., from bacteria or viruses [78,79,146,147]. Likewise, it has difficulty in explaining that other kinases, such as MEKK3 [148] and MEKK1 [149], are also involved in regulating IKK activation. Finally, NEMO may be phosphorylated, ubiquitinated, or sumoylated, and these modifications on NEMO regulate IKK activation [150,151,152].

On the other hand, the structural studies of NEMO–polyubiquitin complexes have provided valuable insights into the mechanism for NEMO regulation of IKK activation. Possibly because of the novelty and potential significance of the finding of NEMO–ubiquitin interaction, the NEMO–polyubiquitin complexes have been subjected to intensive structural studies. More than a dozen reports on the structural characterization of NEMO–polyubiquitin complexes utilizing various means have been published to date. The first batch of studies investigated the NMR or crystal structures of the NEMO UBAN domain in complex with K63-linked or linear diubiquitin. These studies clearly demonstrated that linear diubiquitin has a dramatically higher affinity than K63-linked diubiquitin to NEMO, showed that NEMO forms dimers to interact with diubiquitin, and depicted the interaction orientation and contact sites of the complex components. The hydrophobic patches centered at Ile44 and Phe4 of the distal and proximal parts of diubiquitin, respectively, are critical for the interactions with the UBAN domain. These studies further established, together with then newly found linear ubiquitination of TNFα signaling components, that NEMO’s recognition of linear ubiquitin chains plays a critical role in the IKK/NF-κB pathway [19,44,153]. More recent structural studies demonstrated that the interaction of polyubiquitin chains with NEMO induces NEMO conformational changes [154,155,156,157,158]. These studies further revealed that ubiquitin chain lengths matter in their induction of NEMO conformational changes. The study by Catici et al. utilized rapid-mixing stopped-flow fluorescence spectroscopy to track NEMO conformational change in response to linear ubiquitin chain binding and demonstrated that polyubiquitin binding drives NEMO activity by inducing conformational change to expose hydrophobic residues and, therefore, enhances its affinity for IKKβ and IκBα. This was also verified by another study of theirs using the technology of red edge excitation shift spectroscopy [155]. In addition, these studies suggested that longer ubiquitin chain lengths induce a broader range of accessible NEMO conformational states. It has been suggested that NEMO adopts an auto-inhibited state that is relieved by the binding to M1-linked ubiquitin chains due to the existence of a dynamic “hinge” region (aa112–150) between the N-terminal IKKβ- and C-terminal IκB- binding domains [156]. Also, binding to linear polyubiquitin induces the exposure of a second IKKβ-binding motif in the NEMO ZF, which could stabilize the active NEMO signaling complex and bring IKKβ into the proximity of IκB to catalyze its phosphorylation [157]. Super-resolution microscopy revealed the existence of higher-order NEMO lattice structures that are dependent on the presence of polyubiquitin chains. Such structures may be related to NEMO oligomerization and allow proximity-based trans-autophosphorylation, leading to the cooperative activation of the signaling cascade [159]. Therefore, NEMO is not only an adaptor that functions to assemble complexes, but it could also be a direct IKK activity modulator after interacting with polyubiquitin [157,160].

IKKs were identified as components of remarkably large 700–900 kD complexes [161]. Several recent studies revealed that NEMO–polyubiquitinated substrate interactions trigger liquid–liquid phase separation by forming liquid-like droplets. This could be the basis for the puncta structure observed in cells stimulated with TNFα and IL-1β [42]. NEMO mutations associated with human immunodeficiency abolish its phase separation [42,162]. An intervening domain (IVD) that is conserved in NEMO and optineurin is essential for IKKβ-induced conformational change [160] and also plays an important role in the induction of liquid phase separation [163]. A similar puncta structure called POLKDOTS, which contains ubiquitinated Bcl10, was also observed following TCR stimulation [104]. These studies suggest that polyubiquitin engages NEMO to activate IKK and NF-κB signaling by promoting IKK liquid phase separation.

## 9. NF-κB-Independent Roles of NEMO

Apart from the well-known roles in mediating NF-κB activation and IFN production, other functions of NEMO have also been reported. The first identified NF-κB-independent function of NEMO was its interaction with HIF2α [164]. NEMO is involved in the progression of the metastasis of clear cell renal cell carcinoma by prolonging tumor cell survival via the regulation of apoptosis and activation of the epithelial-to-mesenchymal transition. The oncogenic function of NEMO in renal cell carcinoma is NF-κB-unrelated, but due to its stabilization of HIFα and the activation of the HIF signaling pathway [165]. Mice lacking NEMO in their liver parenchymal cells spontaneously develop steatohepatitis and hepatocellular carcinoma (HCC), and NEMO prevents hepatocarcinogenesis by inhibiting the RIP1 kinase activity-driven hepatocyte apoptosis through NF-κB-dependent and independent functions [166]. Mice with specific deletions of NEMO in their intestinal epithelial cells (IECs) spontaneously develop chronic colitis. However, dysregulation of NF-κB is not responsible for this phenotype. Rather, NEMO prevents intestinal inflammation by inhibiting RIP1 kinase activity-mediated IEC death [167]. Finally, NEMO-deficient podocytes display faster recovery from damage in vivo, and cytoskeletal rearrangements and increased motility of podocytes following in vitro stimulation with IL-1, TNFα, or LPS depend on NEMO. However, this regulation is independent of NF-κB since the expression of an IκB mutant that is resistant to phosphorylation and degradation has no impact on podocyte migration in the presence of IL-1. On the other hand, IL-1 stimulation-induced ERK1/2 phosphorylation is reduced, and RhoA activation is elevated in podocytes with the inhibition of NEMO by siRNA [168]. It would be interesting to investigate whether these NF-κB-independent roles also involve ubiquitin sensing by NEMO.

## 10. Dysfunction in Ubiquitin Binding of NEMO Family of Proteins in Hereditary Diseases

Several hereditary diseases have been linked to NEMO [21]. NEMO mutations are a common hallmark of incontinentia pigmenti and anhidrotic ectodermal dysplasia with immunodeficiency [21]. Patients with EDA-ID, always males with hemizygous NEMO mutations, display life-threatening bacterial and viral infections combined with abnormal development of ectodermal tissues including skin, sweat glands, hair, and teeth. Patients with IP, usually females with heterozygous NEMO mutations, present defects in the neuroectodermal tissues, exhibiting cutaneous lesions and neurological symptoms [169]. The missense mutations in the UBAN domain that cause EDA-ID, including D311N [6,40], D311G [170], E315A [44], and R319Q [171], lead to defects in NEMO’s ubiquitin binding and TNFα- or IL1-induced NF-κB activation. A splice mutation that results in an in-frame deletion (DelQ134-R256) impairs NEMO/SHARPIN interaction and linear ubiquitination and leads to incontinentia pigmenti [172]. Super-resolution microscopy-observed NEMO lattice structures, whose formation relies on interaction with polyubiquitin, are missing from patient cells that contain a genomic rearrangement of exons 4–10 in NEMO and express a truncated protein, the causative of most IP cases [159]. The α-Synuclein aggregates are M1-ubiquitinated, and defects in the ubiquitin-binding of NEMO and NEMO-mediated recruitment of p62 to the α-Synuclein aggregates could be the underlying cause for the neurological symptoms in the patients with IP bearing the NEMO Q330X mutation [86]. The mutation-led reduction in NEMO expression in patients is linked to mycobacteria infection, which sometimes is fatal [173]. Ubiquitination-mediated xenophagy is an effective host means to restrict Mycobacterium tuberculosis, whereas the mycobacterium also exploits virulence factors to dampen host-directed autophagy [174].

Mutations in optineurin have been linked with primary open-angle glaucoma, Paget’s disease of the bone, and amyotrophic lateral sclerosis (ALS) [175]. Q165X, Q398X, Q454E, and E478G (X denotes a stop codon) mutations in optineurin were identified from familial ALS patients. The E478G mutation in optineurin disables its linear ubiquitin chain binding activity and makes the protein fail to suppress TNFα-induced NF-κB activation. In addition, immunohistochemical analyses of motor neurons from patients with optineurin-associated ALS found partial colocalization of linear ubiquitin and activated NF-κB in cytoplasmic inclusions as well as increased active caspases in cytosol. Linear ubiquitination is involved in the pathogenesis of optineurin mutation-associated ALS [176].

These data derived from human genetics studies powerfully decipher the importance of ubiquitin sensing activity of NEMO and optineurin in health and disease. The importance of non-degradative ubiquitination in human diseases is also underscored by the association of mutations in LUBAC components and the deubiquitinases of CYLD, OTULIN, and A20 with various human diseases (see the reviews [11,177,178,179]).

## 11. NEMO Is a Frequent Target of Microbial Virulence Factors

During the course of pathogen–host co-evolution, viruses have obtained the capacity to impede the innate immune response by targeting and neutralizing host proteins [180]. Some viruses depend on their components’ direct interaction with NEMO or their E3 ligase activities to ubiquitinate signaling proteins that subsequently engage NEMO through ubiquitin binding to activate the NF-κB pathway to prime the body’s own innate defense mechanisms against pathogens or elicit pathogenesis. Some pathogens have evolved highly effective counter mechanisms by cleaving NEMO or inducing NEMO degradation. Numerous examples of these have been reported, and some of them can be seen in the review article [21]. Herein, I describe a few newly reported prominent cases, and some of these are also intended to highlight the relevance of ubiquitin-binding activity of NEMO in host defense against virus or viral disease pathogenesis.

The Hepatitis B virus (HBV) e antigen interacts with NEMO and suppresses TRAF6-dependent K63-linked ubiquitination of NEMO to enhance HBV replication and sustain infection [181]. Hepatitis C virus (HCV) NS3 competes with NEMO to interact with LUBAC and inhibits NEMO linear ubiquitination and NF-κB activation to modulate host– antiviral response [182]. The murine cytomegalovirus (Murid herpesvirus 1) M45 protein induces the degradation of NEMO and RIP1 by disrupting their interaction and targeting them individually to selective autophagy (aggrephagy) [183]. Molluscum contagiosum virus, a human-adapted poxvirus, carries two evolved proteins that perturb the activation of NF-κB: MC005 binds to NEMO in a region between the UBAN and IKK binding domains, preventing priming of the IKK complex [184], and MC159 interrupts the interaction between cIAP1 and NEMO and mitigates NEMO polyubiquitination [185]. Arterivirus nsp4 and Feline Infectious Peritonitis Virus Nsp5 both cleave NEMO at multiple sites and inhibit type I IFN production [186,187]. Notably, SARS-CoV-2 has been found to employ several mechanisms to evade antiviral response or augment disease pathogenesis by targeting NEMO. SARS-CoV-2’s main protease M(pro) (also termed 3CL^pro^) cleaves NEMO at Q231 to separate the IKK binding domain and the ubiquitin-binding domain and disables NEMO’s function, leading to string vessels in the brain, which is considered as the major cause for COVID-19 patients’ neurological symptoms [188,189]. SARS-CoV-2 ORF9b inhibits RIG-I-MAVS’s antiviral signaling by interrupting K63-linked ubiquitination of NEMO and, subsequently, reduces IFN production [190]. K63-linked ubiquitin chains on SARS-CoV-2 NSP6 and ORF7a and linear ubiquitin chains on SARS-CoV-2 NSP14 recruit NEMO to activate NF-κB, increasing COVID-19 disease severity by enhancing inflammation [147,191]. As discussed above, viral factors, such as the HTLV-1 Tax protein, can also utilize NEMO’s ubiquitin sensing activity to induce cancer.

Several studies also reported viral targeting of optineurin. The NS3 protein of the Bluetongue virus binds and neutralizes optineurin’s sensing of ubiquitinated TBK1 at the Golgi apparatus, thus diminishing TBK1 activation and the ensuing IFN production [69]. The Seneca Valley virus 3C protease cleaves optineurin to impair selective autophagy and type I interferon production of host cells [192].

## 12. Conclusive Remarks and Perspectives

After the finding of NEMO as a ubiquitin-binding protein, we have witnessed enormous progress in understanding mechanistically how IKK is activated by diverse signaling pathways that use distinct sets of intermediaries in propagating signals from a variety of membrane and intracellular receptors, environmental cues, and intracellular stressors (Figure 2 and Figure 3). NEMO also involves ubiquitin binding in promoting type I IFN production that is critical for antiviral response. This review has summarized the advances in understanding of how NEMO acts as a decoder in transmitting non-degradative ubiquitin signals embodied in diverse topologies of ubiquitin chains that are attached to signaling intermediaries to IKK activation and IFN production. These molecular events impact a variety of cellular and organismic responses crucial for health maintenance. Interestingly, NEMO is a frequent target of virulence factors from many types of viruses and bacteria, being used by microbes to induce pathogenesis, elicit host response, or develop counter mechanisms to evade host defense. Optineurin engages non-degradative ubiquitin chains to play an important role in autophagy and in regulating NF-κB activation and IFN production, and thus immune responses and antiviral responses. The dysfunction of optineurin is associated with neurological diseases. Tremendous progress has been made on the understanding of the molecular bases and functions of the NEMO family of protein in non-degenerative ubiquitin signaling in the last two decades.

However, some questions remain to be answered. An important question is how the NEMO family of proteins recognize ubiquitinated substrates to achieve signaling specificity that dictates distinct functional outcomes, as they possess similar ubiquitin binding activities. One possibility is that their direct weak interactions with the substrates might contribute to the specific recognition of an ubiquitinated protein [158]. NEMO binds different linkages and lengths of ubiquitin chains with differential affinities, which may explain quantitatively distinct NF-κB activation patterns in response to diverse cell stimuli [22]. Finally, the relevant signaling events might need to occur at specific cellular locations. For instance, IKK and TBK1 are activated by MVAS aggregates at the mitochondrial membrane [52]. Further biochemistry and structural studies equipped with more powerful methodologies, in particular sophisticated imaging of multiple protein complexes, may help to depict a clearer picture.

NEMO, optineurin, and ABIN1 are all involved in regulating signaling and autophagy. Optineurin and ABIN1 may inhibit NEMO-mediated NF-κB activation by competing with NEMO for ubiquitinated signaling molecules. On the other hand, optineurin, NEMO, and ABIN1 all play a positive role in selective autophagy. Damaged mitochondria recruit both optineurin and NEMO via their ubiquitinated membrane components. However, the mitochondria that recruit NEMO do not overlap with the population that interacts with optineurin, presenting distinct consequences: NF-κB activation and mitophagy, respectively [83]. How these intriguing interplays happen remains to be elucidated.

There might be a connection between NEMO-mediated NF-κB signaling and selective autophagy. K63-linked or linear ubiquitinated Bcl10 engages NEMO for both TCR engagement-induced NF-κB activation and Bcl10 autophagic degradation [93,104]. These occurrences require the formation of intracellular puncta structures in which Bcl10 is localized after TCR activation [104]. Likely as reported for TNFR1 signaling and IL-1R signaling, the TCR activation-mediated puncta structures result from protein aggregation-relevant liquid phase separation. An attractive scenario is that the selective autophagy is utilized to clear the TCR activation-led signaling protein aggregates, whose accumulation would eventually be harmful to cells (Figure 3). Also, selective autophagy may serve as a means for signaling termination that usually ensues the signaling activation. This interplay may constitute a fine mechanism for modulating the strength and duration of T cell signaling and, as such, is critical for immunological fitness. The autophagy receptor p62 also binds K63-linked polyubiquitinated protein substrates to trigger the formation of liquid phase separation structures known as the p62 bodies, which serve as a platform for forming autophagosomes that deliver cargo proteins to lysosomes for degradation and for eliciting anti-oxidative stress response [193,194]. In this sense, the so-called “non-degradative ubiquitination”, such as K63-linked and linear ubiquitination that mediates signaling in the first place, may be eventually involved in protein degradation, though in lysosomes rather than proteasomes.

Non-proteolytic ubiquitination regulates a broad range of cellular processes, and the dysregulation of the NEMO family of proteins as ubiquitin receptors is linked with the development of multiple human diseases, including immunodeficiency, neurodegenerative diseases, and cancer. Additionally, NEMO has a few NF-κB-independent roles. Further studies on NEMO’s NF-κB independent roles and the roles of other less characterized NEMO family of proteins may lead to discoveries of new functions for non-degradative ubiquitination. Recent findings indicate that CEP55, via interacting with polyubiquitin, plays an important role in cell division, and defects in CEP55′s ubiquitin-binding activity may be an underlying mechanism for CEP55 mutation-caused neurological disorders [10]. Considering the importance of the NEMO family of proteins as polyubiquitin receptors in cellular signaling, profound molecular understanding of relevant signaling events will facilitate the design of targeted strategies to treat non-degradative ubiquitination dysregulation-caused diseases, such as inflammatory conditions, immunity disorders, and neurodegenerative diseases, for which endeavors are already ongoing [195,196].

## Data Availability

Not applicable.

## Abbreviations

ALS: amyotrophic lateral sclerosis; CEP55: centrosomal protein 55 kDa; CBM: CARMA1-Bcl10-MALT1; DSB: double strand break; DUB: deubiquitinase; E1: ubiquitin-activating enzymes; E2: ubiquitin-conjugating enzyme; E3: ubiquitin ligase; EDA-ID: anhidrotic ectodermal dysplasia with immunodeficiency; HBV: hepatitis B virus; HCV: hepatitis C virus; IEC: intestinal epithelial cells; IFN: interferon; IKK: IκB kinase; IP: incontinentia pigmenti; K: lysine; LPS: lipopolysaccharide; LUBAC: linear ubiquitin chain assembly complex; MALT: mucosa-associated lymphoid tissue; MOMP: mitochondrial outer membrane permeabilization; NEMO: NF-κB-essential modulator; OPTN: optineurin; PAMP: pathogen-associated molecular pattern; PTM: post-translational modification; PRR: pattern-recognition receptor; RIP1: receptor interaction protein 1; RLR: RIG-I-like receptor; TCR: T cell receptor; TIR: Toll/interleukin-1 receptor; TLR: toll-like receptor; TNF: tumor necrosis factor; TRAF: Tumor necrosis factor receptor-associated factor; Treg: regulatory T cell; Ub: ubiquitin; UBAN: ubiquitin binding in ABIN and NEMO.

## References

[1] Hayden M.S., Ghosh S. (2004). Signaling to NF-kappaB. Genes Dev..

[2] Silverman N., Maniatis T. (2001). NF-kappaB signaling pathways in mammalian and insect innate immunity. Genes Dev..

[3] Mercurio F., Zhu H., Murray B.W., Shevchenko A., Bennett B.L., Li J., Young D.B., Barbosa M., Mann M., Manning A. (1997). IKK-1 and IKK-2: Cytokine-activated IkappaB kinases essential for NF-kappaB activation. Science.

[4] Rothwarf D.M., Zandi E., Natoli G., Karin M. (1998). IKK-gamma is an essential regulatory subunit of the IkappaB kinase complex. Nature.

[5] Hayden M.S., Ghosh S. (2008). Shared principles in NF-kappaB signaling. Cell.

[6] Wu C.J., Conze D.B., Li T., Srinivasula S.M., Ashwell J.D. (2006). Sensing of Lys 63-linked polyubiquitination by NEMO is a key event in NF-kappaB activation [corrected]. Nat. Cell Biol..

[7] Ea C.K., Deng L., Xia Z.P., Pineda G., Chen Z.J. (2006). Activation of IKK by TNFalpha requires site-specific ubiquitination of RIP1 and polyubiquitin binding by NEMO. Mol. Cell.

[8] Zhu G., Wu C.J., Zhao Y., Ashwell J.D. (2007). Optineurin negatively regulates TNFalpha- induced NF-kappaB activation by competing with NEMO for ubiquitinated RIP. Curr. Biol..

[9] Wagner S., Carpentier I., Rogov V., Kreike M., Ikeda F., Lohr F., Wu C.J., Ashwell J.D., Dotsch V., Dikic I. (2008). Ubiquitin binding mediates the NF-kappaB inhibitory potential of ABIN proteins. Oncogene.

[10] Said Halidi K.N., Fontan E., Boucharlat A., Davignon L., Charpentier M., Boulle M., Weil R., Israel A., Laplantine E., Agou F. (2019). Two NEMO-like Ubiquitin-Binding Domains in CEP55 Differently Regulate Cytokinesis. iScience.

[11] Oikawa D., Sato Y., Ito H., Tokunaga F. (2020). Linear Ubiquitin Code: Its Writer, Erasers, Decoders, Inhibitors, and Implications in Disorders. Int. J. Mol. Sci..

[12] Hershko A., Ciechanover A. (1998). The ubiquitin system. Annu. Rev. Biochem..

[13] Hicke L. (2001). Protein regulation by monoubiquitin. Nat. Rev. Mol. Cell Biol..

[14] Mulder M.P.C., Witting K.F., Ovaa H. (2020). Cracking the Ubiquitin Code: The Ubiquitin Toolbox. Curr. Issues Mol. Biol..

[15] Komander D., Rape M. (2012). The ubiquitin code. Annu. Rev. Biochem..

[16] Komander D. (2009). The emerging complexity of protein ubiquitination. Biochem. Soc. Trans..

[17] Husnjak K., Dikic I. (2012). Ubiquitin-binding proteins: Decoders of ubiquitin-mediated cellular functions. Annu. Rev. Biochem..

[18] Clark K., Nanda S., Cohen P. (2013). Molecular control of the NEMO family of ubiquitin-binding proteins. Nat. Rev. Mol. Cell Biol..

[19] Rahighi S., Ikeda F., Kawasaki M., Akutsu M., Suzuki N., Kato R., Kensche T., Uejima T., Bloor S., Komander D. (2009). Specific recognition of linear ubiquitin chains by NEMO is important for NF-kappaB activation. Cell.

[20] Cordier F., Grubisha O., Traincard F., Veron M., Delepierre M., Agou F. (2009). The zinc finger of NEMO is a functional ubiquitin-binding domain. J. Biol. Chem..

[21] Maubach G., Schmadicke A.C., Naumann M. (2017). NEMO Links Nuclear Factor-kappaB to Human Diseases. Trends Mol. Med..

[22] Kensche T., Tokunaga F., Ikeda F., Goto E., Iwai K., Dikic I. (2012). Analysis of nuclear factor-kappaB (NF-kappaB) essential modulator (NEMO) binding to linear and lysine-linked ubiquitin chains and its role in the activation of NF-kappaB. J. Biol. Chem..

[23] Dynek J.N., Goncharov T., Dueber E.C., Fedorova A.V., Izrael-Tomasevic A., Phu L., Helgason E., Fairbrother W.J., Deshayes K., Kirkpatrick D.S. (2010). c-IAP1 and UbcH5 promote K11-linked polyubiquitination of RIP1 in TNF signalling. EMBO J..

[24] Kist M., Komuves L.G., Goncharov T., Dugger D.L., Yu C., Roose-Girma M., Newton K., Webster J.D., Vucic D. (2021). Impaired RIPK1 ubiquitination sensitizes mice to TNF toxicity and inflammatory cell death. Cell Death Differ..

[25] Liu J., Han C., Xie B., Wu Y., Liu S., Chen K., Xia M., Zhang Y., Song L., Li Z. (2014). Rhbdd3 controls autoimmunity by suppressing the production of IL-6 by dendritic cells via K27-linked ubiquitination of the regulator NEMO. Nat. Immunol..

[26] Grou C.P., Pinto M.P., Mendes A.V., Domingues P., Azevedo J.E. (2015). The de novo synthesis of ubiquitin: Identification of deubiquitinases acting on ubiquitin precursors. Sci. Rep..

[27] Kirisako T., Kamei K., Murata S., Kato M., Fukumoto H., Kanie M., Sano S., Tokunaga F., Tanaka K., Iwai K. (2006). A ubiquitin ligase complex assembles linear polyubiquitin chains. EMBO J..

[28] Tokunaga F., Sakata S., Saeki Y., Satomi Y., Kirisako T., Kamei K., Nakagawa T., Kato M., Murata S., Yamaoka S. (2009). Involvement of linear polyubiquitylation of NEMO in NF-kappaB activation. Nat. Cell Biol..

[29] Ohtake F. (2022). Branched ubiquitin code: From basic biology to targeted protein degradation. J. Biochem..

[30] Emmerich C.H., Ordureau A., Strickson S., Arthur J.S., Pedrioli P.G., Komander D., Cohen P. (2013). Activation of the canonical IKK complex by K63/M1-linked hybrid ubiquitin chains. Proc. Natl. Acad. Sci. USA.

[31] Ohtake F., Saeki Y., Ishido S., Kanno J., Tanaka K. (2016). The K48-K63 Branched Ubiquitin Chain Regulates NF-kappaB Signaling. Mol. Cell.

[32] Dikic I., Schulman B.A. (2023). An expanded lexicon for the ubiquitin code. Nat. Rev. Mol. Cell Biol..

[33] Kelsall I.R., Zhang J., Knebel A., Arthur J.S.C., Cohen P. (2019). The E3 ligase HOIL-1 catalyses ester bond formation between ubiquitin and components of the Myddosome in mammalian cells. Proc. Natl. Acad. Sci. USA.

[34] Akira S., Uematsu S., Takeuchi O. (2006). Pathogen recognition and innate immunity. Cell.

[35] Cai X., Chiu Y.H., Chen Z.J. (2014). The cGAS-cGAMP-STING pathway of cytosolic DNA sensing and signaling. Mol. Cell.

[36] Janeway C.A., Medzhitov R. (2002). Innate immune recognition. Annu. Rev. Immunol..

[37] Newton K., Dixit V.M. (2012). Signaling in innate immunity and inflammation. Cold Spring Harb. Perspect. Biol..

[38] Blasius A.L., Beutler B. (2010). Intracellular toll-like receptors. Immunity.

[39] Conze D.B., Wu C.J., Thomas J.A., Landstrom A., Ashwell J.D. (2008). Lys63-linked polyubiquitination of IRAK-1 is required for interleukin-1 receptor- and toll-like receptor-mediated NF-kappaB activation. Mol. Cell Biol..

[40] Windheim M., Stafford M., Peggie M., Cohen P. (2008). Interleukin-1 (IL-1) induces the Lys63-linked polyubiquitination of IL-1 receptor-associated kinase 1 to facilitate NEMO binding and the activation of IkappaBalpha kinase. Mol. Cell Biol..

[41] Kanayama A., Seth R.B., Sun L., Ea C.K., Hong M., Shaito A., Chiu Y.H., Deng L., Chen Z.J. (2004). TAB2 and TAB3 activate the NF-kappaB pathway through binding to polyubiquitin chains. Mol. Cell.

[42] Du M., Ea C.K., Fang Y., Chen Z.J. (2022). Liquid phase separation of NEMO induced by polyubiquitin chains activates NF-kappaB. Mol. Cell.

[43] Cohen P., Strickson S. (2017). The role of hybrid ubiquitin chains in the MyD88 and other innate immune signalling pathways. Cell Death Differ..

[44] Lo Y.C., Lin S.C., Rospigliosi C.C., Conze D.B., Wu C.J., Ashwell J.D., Eliezer D., Wu H. (2009). Structural basis for recognition of diubiquitins by NEMO. Mol. Cell.

[45] Cusson-Hermance N., Khurana S., Lee T.H., Fitzgerald K.A., Kelliher M.A. (2005). Rip1 mediates the Trif-dependent toll-like receptor 3- and 4-induced NF-kappaB activation but does not contribute to interferon regulatory factor 3 activation. J. Biol. Chem..

[46] Zinngrebe J., Rieser E., Taraborrelli L., Peltzer N., Hartwig T., Ren H., Kovacs I., Endres C., Draber P., Darding M. (2016). LUBAC deficiency perturbs TLR3 signaling to cause immunodeficiency and autoinflammation. J. Exp. Med..

[47] Chen J., Chen Z.J. (2013). Regulation of NF-kappaB by ubiquitination. Curr. Opin. Immunol..

[48] Nanda S.K., Venigalla R.K., Ordureau A., Patterson-Kane J.C., Powell D.W., Toth R., Arthur J.S., Cohen P. (2011). Polyubiquitin binding to ABIN1 is required to prevent autoimmunity. J. Exp. Med..

[49] Vajjhala P.R., Ve T., Bentham A., Stacey K.J., Kobe B. (2017). The molecular mechanisms of signaling by cooperative assembly formation in innate immunity pathways. Mol. Immunol..

[50] Seth R.B., Sun L., Ea C.K., Chen Z.J. (2005). Identification and characterization of MAVS, a mitochondrial antiviral signaling protein that activates NF-kappaB and IRF 3. Cell.

[51] Rehwinkel J., Gack M.U. (2020). RIG-I-like receptors: Their regulation and roles in RNA sensing. Nat. Rev. Immunol..

[52] Hou F., Sun L., Zheng H., Skaug B., Jiang Q.X., Chen Z.J. (2011). MAVS forms functional prion-like aggregates to activate and propagate antiviral innate immune response. Cell.

[53] Perales-Linares R., Navas-Martin S. (2013). Toll-like receptor 3 in viral pathogenesis: Friend or foe?. Immunology.

[54] Audry M., Ciancanelli M., Yang K., Cobat A., Chang H.H., Sancho-Shimizu V., Lorenzo L., Niehues T., Reichenbach J., Li X.X. (2011). NEMO is a key component of NF-kappaB- and IRF-3-dependent TLR3-mediated immunity to herpes simplex virus. J. Allergy Clin. Immunol..

[55] Zhao T., Yang L., Sun Q., Arguello M., Ballard D.W., Hiscott J., Lin R. (2007). The NEMO adaptor bridges the nuclear factor-kappaB and interferon regulatory factor signaling pathways. Nat. Immunol..

[56] Zeng W., Xu M., Liu S., Sun L., Chen Z.J. (2009). Key role of Ubc5 and lysine-63 polyubiquitination in viral activation of IRF3. Mol. Cell.

[57] Ran Y., Zhang J., Liu L.L., Pan Z.Y., Nie Y., Zhang H.Y., Wang Y.Y. (2016). Autoubiquitination of TRIM26 links TBK1 to NEMO in RLR-mediated innate antiviral immune response. J. Mol. Cell Biol..

[58] Liu B., Zhang M., Chu H., Zhang H., Wu H., Song G., Wang P., Zhao K., Hou J., Wang X. (2017). The ubiquitin E3 ligase TRIM31 promotes aggregation and activation of the signaling adaptor MAVS through Lys63-linked polyubiquitination. Nat. Immunol..

[59] Hou J., Han L., Zhao Z., Liu H., Zhang L., Ma C., Yi F., Liu B., Zheng Y., Gao C. (2021). USP18 positively regulates innate antiviral immunity by promoting K63-linked polyubiquitination of MAVS. Nat. Commun..

[60] Castanier C., Zemirli N., Portier A., Garcin D., Bidere N., Vazquez A., Arnoult D. (2012). MAVS ubiquitination by the E3 ligase TRIM25 and degradation by the proteasome is involved in type I interferon production after activation of the antiviral RIG-I-like receptors. BMC Biol..

[61] Gack M.U., Shin Y.C., Joo C.H., Urano T., Liang C., Sun L., Takeuchi O., Akira S., Chen Z., Inoue S. (2007). TRIM25 RING-finger E3 ubiquitin ligase is essential for RIG-I-mediated antiviral activity. Nature.

[62] Lee N.R., Kim H.I., Choi M.S., Yi C.M., Inn K.S. (2015). Regulation of MDA5-MAVS Antiviral Signaling Axis by TRIM25 through TRAF6-Mediated NF-kappaB Activation. Mol. Cells.

[63] Inn K.S., Gack M.U., Tokunaga F., Shi M., Wong L.Y., Iwai K., Jung J.U. (2011). Linear ubiquitin assembly complex negatively regulates RIG-I- and TRIM25-mediated type I interferon induction. Mol. Cell.

[64] MacDuff D.A., Baldridge M.T., Qaqish A.M., Nice T.J., Darbandi A.D., Hartley V.L., Peterson S.T., Miner J.J., Iwai K., Virgin H.W. (2018). HOIL1 Is Essential for the Induction of Type I and III Interferons by MDA5 and Regulates Persistent Murine Norovirus Infection. J. Virol..

[65] Belgnaoui S.M., Paz S., Samuel S., Goulet M.L., Sun Q., Kikkert M., Iwai K., Dikic I., Hiscott J., Lin R. (2012). Linear ubiquitination of NEMO negatively regulates the interferon antiviral response through disruption of the MAVS-TRAF3 complex. Cell Host Microbe.

[66] Mankouri J., Fragkoudis R., Richards K.H., Wetherill L.F., Harris M., Kohl A., Elliott R.M., Macdonald A. (2010). Optineurin negatively regulates the induction of IFNbeta in response to RNA virus infection. PLoS Pathog..

[67] Gleason C.E., Ordureau A., Gourlay R., Arthur J.S.C., Cohen P. (2011). Polyubiquitin binding to optineurin is required for optimal activation of TANK-binding kinase 1 and production of interferon beta. J. Biol. Chem..

[68] Outlioua A., Pourcelot M., Arnoult D. (2018). The Role of Optineurin in Antiviral Type I Interferon Production. Front. Immunol..

[69] Pourcelot M., Zemirli N., Silva Da Costa L., Loyant R., Garcin D., Vitour D., Munitic I., Vazquez A., Arnoult D. (2016). The Golgi apparatus acts as a platform for TBK1 activation after viral RNA sensing. BMC Biol..

[70] Munitic I., Giardino Torchia M.L., Meena N.P., Zhu G., Li C.C., Ashwell J.D. (2013). Optineurin insufficiency impairs IRF3 but not NF-kappaB activation in immune cells. J. Immunol..

[71] Slowicka K., Vereecke L., Mc Guire C., Sze M., Maelfait J., Kolpe A., Saelens X., Beyaert R., van Loo G. (2016). Optineurin deficiency in mice is associated with increased sensitivity to Salmonella but does not affect proinflammatory NF-kappaB signaling. Eur. J. Immunol..

[72] Wu X., Wang Y., Chen B., Liu Y., Li F., Ou Y., Zhang H., Wu X., Li X., Wang L. (2024). ABIN1 (Q478) is Required to Prevent Hematopoietic Deficiencies through Regulating Type I IFNs Expression. Adv. Sci..

[73] Zaffagnini G., Martens S. (2016). Mechanisms of Selective Autophagy. J. Mol. Biol..

[74] Wild P., Farhan H., McEwan D.G., Wagner S., Rogov V.V., Brady N.R., Richter B., Korac J., Waidmann O., Choudhary C. (2011). Phosphorylation of the autophagy receptor optineurin restricts *Salmonella* growth. Science.

[75] Qiu Y., Wang J., Li H., Yang B., Wang J., He Q., Weng Q. (2022). Emerging views of OPTN (optineurin) function in the autophagic process associated with disease. Autophagy.

[76] Ames J., Yadavalli T., Suryawanshi R., Hopkins J., Agelidis A., Patil C., Fredericks B., Tseng H., Valyi-Nagy T., Shukla D. (2021). OPTN is a host intrinsic restriction factor against neuroinvasive HSV-1 infection. Nat. Commun..

[77] Patil C.D., Shukla D. (2022). OPTN (optineurin)-mediated selective autophagy prevents neurodegeneration due to herpesvirus infection. Autophagy.

[78] Noad J., von der Malsburg A., Pathe C., Michel M.A., Komander D., Randow F. (2017). LUBAC-synthesized linear ubiquitin chains restrict cytosol-invading bacteria by activating autophagy and NF-kappaB. Nat. Microbiol..

[79] van Wijk S.J.L., Fricke F., Herhaus L., Gupta J., Hotte K., Pampaloni F., Grumati P., Kaulich M., Sou Y.S., Komatsu M. (2017). Linear ubiquitination of cytosolic *Salmonella* Typhimurium activates NF-kappaB and restricts bacterial proliferation. Nat. Microbiol..

[80] Budzik J.M., Swaney D.L., Jimenez-Morales D., Johnson J.R., Garelis N.E., Repasy T., Roberts A.W., Popov L.M., Parry T.J., Pratt D. (2020). Dynamic post-translational modification profiling of *Mycobacterium tuberculosis*-infected primary macrophages. Elife.

[81] Zhang R., Varela M., Vallentgoed W., Forn-Cuni G., van der Vaart M., Meijer A.H. (2019). The selective autophagy receptors Optineurin and p62 are both required for zebrafish host resistance to mycobacterial infection. PLoS Pathog..

[82] Ordureau A., Heo J.M., Duda D.M., Paulo J.A., Olszewski J.L., Yanishevski D., Rinehart J., Schulman B.A., Harper J.W. (2015). Defining roles of PARKIN and ubiquitin phosphorylation by PINK1 in mitochondrial quality control using a ubiquitin replacement strategy. Proc. Natl. Acad. Sci. USA.

[83] Harding O., Holzer E., Riley J.F., Martens S., Holzbaur E.L.F. (2023). Damaged mitochondria recruit the effector NEMO to activate NF-kappaB signaling. Mol. Cell.

[84] Eapen V.V., Swarup S., Hoyer M.J., Paulo J.A., Harper J.W. (2021). Quantitative proteomics reveals the selectivity of ubiquitin-binding autophagy receptors in the turnover of damaged lysosomes by lysophagy. Elife.

[85] Shaid S., Brandts C.H., Serve H., Dikic I. (2013). Ubiquitination and selective autophagy. Cell Death Differ..

[86] Furthmann N., Bader V., Angersbach L., Blusch A., Goel S., Sanchez-Vicente A., Krause L.J., Chaban S.A., Grover P., Trinkaus V.A. (2023). NEMO reshapes the alpha-Synuclein aggregate interface and acts as an autophagy adapter by co-condensation with p62. Nat. Commun..

[87] Merline R., Rodig H., Zeng-Brouwers J., Poluzzi C., Tascher G., Michaelis J., Lopez-Mosqueda J., Rhiner A., Huber L.S., Diehl V. (2023). A20 binding and inhibitor of nuclear factor kappa B (NF-kappaB)-1 (ABIN-1): A novel modulator of mitochondrial autophagy. Am. J. Physiol. Cell Physiol..

[88] Hayden M.S., West A.P., Ghosh S. (2006). NF-kappaB and the immune response. Oncogene.

[89] Courtney A.H., Lo W.L., Weiss A. (2018). TCR Signaling: Mechanisms of Initiation and Propagation. Trends Biochem. Sci..

[90] Clements J.L., Boerth N.J., Lee J.R., Koretzky G.A. (1999). Integration of T cell receptor-dependent signaling pathways by adapter proteins. Annu. Rev. Immunol..

[91] Weil R., Israel A. (2006). Deciphering the pathway from the TCR to NF-kappaB. Cell Death Differ..

[92] Oeckinghaus A., Wegener E., Welteke V., Ferch U., Arslan S.C., Ruland J., Scheidereit C., Krappmann D. (2007). Malt1 ubiquitination triggers NF-kappaB signaling upon T-cell activation. EMBO J..

[93] Wu C.J., Ashwell J.D. (2008). NEMO recognition of ubiquitinated Bcl10 is required for T cell receptor-mediated NF-kappaB activation. Proc. Natl. Acad. Sci. USA.

[94] Yamamoto M., Sato S., Saitoh T., Sakurai H., Uematsu S., Kawai T., Ishii K.J., Takeuchi O., Akira S. (2006). Cutting Edge: Pivotal function of Ubc13 in thymocyte TCR signaling. J. Immunol..

[95] Yang Y., Kelly P., Shaffer A.L., Schmitz R., Yoo H.M., Liu X., Huang D.W., Webster D., Young R.M., Nakagawa M. (2016). Targeting Non-proteolytic Protein Ubiquitination for the Treatment of Diffuse Large B Cell Lymphoma. Cancer Cell.

[96] Blonska M., Pappu B.P., Matsumoto R., Li H., Su B., Wang D., Lin X. (2007). The CARMA1-Bcl10 signaling complex selectively regulates JNK2 kinase in the T cell receptor-signaling pathway. Immunity.

[97] Dubois S.M., Alexia C., Wu Y., Leclair H.M., Leveau C., Schol E., Fest T., Tarte K., Chen Z.J., Gavard J. (2014). A catalytic-independent role for the LUBAC in NF-kappaB activation upon antigen receptor engagement and in lymphoma cells. Blood.

[98] Yang Y.K., Yang C., Chan W., Wang Z., Deibel K.E., Pomerantz J.L. (2016). Molecular Determinants of Scaffold-induced Linear Ubiquitinylation of B Cell Lymphoma/Leukemia 10 (Bcl10) during T Cell Receptor and Oncogenic Caspase Recruitment Domain-containing Protein 11 (CARD11) Signaling. J. Biol. Chem..

[99] Chang J.H., Xiao Y., Hu H., Jin J., Yu J., Zhou X., Wu X., Johnson H.M., Akira S., Pasparakis M. (2012). Ubc13 maintains the suppressive function of regulatory T cells and prevents their conversion into effector-like T cells. Nat. Immunol..

[100] Redecke V., Chaturvedi V., Kuriakose J., Hacker H. (2016). SHARPIN controls the development of regulatory T cells. Immunology.

[101] Teh C.E., Lalaoui N., Jain R., Policheni A.N., Heinlein M., Alvarez-Diaz S., Sheridan J.M., Rieser E., Deuser S., Darding M. (2016). Linear ubiquitin chain assembly complex coordinates late thymic T-cell differentiation and regulatory T-cell homeostasis. Nat. Commun..

[102] Zhao Y., Thornton A.M., Kinney M.C., Ma C.A., Spinner J.J., Fuss I.J., Shevach E.M., Jain A. (2011). The deubiquitinase CYLD targets Smad7 protein to regulate transforming growth factor beta (TGF-beta) signaling and the development of regulatory T cells. J. Biol. Chem..

[103] Montecalvo A., Watkins S.C., Orange J., Kane L.P. (2017). Inducible turnover of optineurin regulates T cell activation. Mol. Immunol..

[104] Paul S., Kashyap A.K., Jia W., He Y.W., Schaefer B.C. (2012). Selective autophagy of the adaptor protein Bcl10 modulates T cell receptor activation of NF-kappaB. Immunity.

[105] Liu T., Zhang L., Joo D., Sun S.C. (2017). NF-kappaB signaling in inflammation. Signal Transduct. Target. Ther..

[106] Aggarwal B.B. (2003). Signalling pathways of the TNF superfamily: A double-edged sword. Nat. Rev. Immunol..

[107] van Loo G., Bertrand M.J.M. (2023). Death by TNF: A road to inflammation. Nat. Rev. Immunol..

[108] Wajant H., Pfizenmaier K., Scheurich P. (2003). Tumor necrosis factor signaling. Cell Death Differ..

[109] Mahoney D.J., Cheung H.H., Mrad R.L., Plenchette S., Simard C., Enwere E., Arora V., Mak T.W., Lacasse E.C., Waring J. (2008). Both cIAP1 and cIAP2 regulate TNFalpha-mediated NF-kappaB activation. Proc. Natl. Acad. Sci. USA.

[110] Varfolomeev E., Goncharov T., Fedorova A.V., Dynek J.N., Zobel K., Deshayes K., Fairbrother W.J., Vucic D. (2008). c-IAP1 and c-IAP2 are critical mediators of tumor necrosis factor alpha (TNFalpha)-induced NF-kappaB activation. J. Biol. Chem..

[111] Haas T.L., Emmerich C.H., Gerlach B., Schmukle A.C., Cordier S.M., Rieser E., Feltham R., Vince J., Warnken U., Wenger T. (2009). Recruitment of the linear ubiquitin chain assembly complex stabilizes the TNF-R1 signaling complex and is required for TNF-mediated gene induction. Mol. Cell.

[112] Emmerich C.H., Schmukle A.C., Walczak H. (2011). The emerging role of linear ubiquitination in cell signaling. Sci. Signal.

[113] Witt A., Vucic D. (2017). Diverse ubiquitin linkages regulate RIP kinases-mediated inflammatory and cell death signaling. Cell Death Differ..

[114] Zhang J., Clark K., Lawrence T., Peggie M.W., Cohen P. (2014). An unexpected twist to the activation of IKKbeta: TAK1 primes IKKbeta for activation by autophosphorylation. Biochem. J..

[115] Kaneko N., Kurata M., Yamamoto T., Morikawa S., Masumoto J. (2019). The role of interleukin-1 in general pathology. Inflamm. Regen..

[116] Rodgers M.A., Bowman J.W., Fujita H., Orazio N., Shi M., Liang Q., Amatya R., Kelly T.J., Iwai K., Ting J. (2014). The linear ubiquitin assembly complex (LUBAC) is essential for NLRP3 inflammasome activation. J. Exp. Med..

[117] Vringer E., Heilig R., Riley J.S., Black A., Cloix C., Skalka G., Montes-Gomez A.E., Aguado A., Lilla S., Walczak H. (2024). Mitochondrial outer membrane integrity regulates a ubiquitin-dependent and NF-kappaB-mediated inflammatory response. EMBO J..

[118] Heyninck K., Kreike M.M., Beyaert R. (2003). Structure-function analysis of the A20-binding inhibitor of NF-kappa B activation, ABIN-1. FEBS Lett..

[119] El Bakkouri K., Wullaert A., Haegman M., Heyninck K., Beyaert R. (2005). Adenoviral gene transfer of the NF-kappa B inhibitory protein ABIN-1 decreases allergic airway inflammation in a murine asthma model. J. Biol. Chem..

[120] Dziedzic S.A., Su Z., Jean Barrett V., Najafov A., Mookhtiar A.K., Amin P., Pan H., Sun L., Zhu H., Ma A. (2018). ABIN-1 regulates RIPK1 activation by linking Met1 ubiquitylation with Lys63 deubiquitylation in TNF-RSC. Nat. Cell Biol..

[121] Jiang H., Xie Y., Hu Z., Lu J., Zhang J., Li H., Zeng K., Peng W., Yang C., Huang J. (2025). VANGL2 alleviates inflammatory bowel disease by recruiting the ubiquitin ligase MARCH8 to limit NLRP3 inflammasome activation through OPTN-mediated selective autophagy. PLoS Biol..

[122] Yang Y., Xia F., Hermance N., Mabb A., Simonson S., Morrissey S., Gandhi P., Munson M., Miyamoto S., Kelliher M.A. (2011). A cytosolic ATM/NEMO/RIP1 complex recruits TAK1 to mediate the NF-kappaB and p38 mitogen-activated protein kinase (MAPK)/MAPK-activated protein 2 responses to DNA damage. Mol. Cell. Biol..

[123] Zhao H., Zhu M., Dou G., Zhao H., Zhu B., Li J., Liao J., Xu X. (2014). BCL10 regulates RNF8/RNF168-mediated ubiquitination in the DNA damage response. Cell Cycle.

[124] Niu J., Shi Y., Iwai K., Wu Z.H. (2011). LUBAC regulates NF-kappaB activation upon genotoxic stress by promoting linear ubiquitination of NEMO. EMBO J..

[125] Xia Y., Shen S., Verma I.M. (2014). NF-kappaB, an active player in human cancers. Cancer Immunol. Res..

[126] Massoumi R. (2011). CYLD: A deubiquitination enzyme with multiple roles in cancer. Future Oncol..

[127] Hymowitz S.G., Wertz I.E. (2010). A20: From ubiquitin editing to tumour suppression. Nat. Rev. Cancer.

[128] Song Z., Wei W., Xiao W., Al-Saleem E.D., Nejati R., Chen L., Yin J., Fabrizio J., Petrus M.N., Waldmann T.A. (2020). Essential role of the linear ubiquitin chain assembly complex and TAK1 kinase in A20 mutant Hodgkin lymphoma. Proc. Natl. Acad. Sci. USA.

[129] Sun S.C., Yamaoka S. (2005). Activation of NF-kappaB by HTLV-I and implications for cell transformation. Oncogene.

[130] Lavorgna A., Harhaj E.W. (2014). Regulation of HTLV-1 tax stability, cellular trafficking and NF-kappaB activation by the ubiquitin-proteasome pathway. Viruses.

[131] Song L., Gong H., Lin C., Wang C., Liu L., Wu J., Li M., Li J. (2012). Flotillin-1 promotes tumor necrosis factor-alpha receptor signaling and activation of NF-kappaB in esophageal squamous cell carcinoma cells. Gastroenterology.

[132] Song K., Cai X., Dong Y., Wu H., Wei Y., Shankavaram U.T., Cui K., Lee Y., Zhu B., Bhattacharjee S. (2021). Epsins 1 and 2 promote NEMO linear ubiquitination via LUBAC to drive breast cancer development. J. Clin. Invest..

[133] Dai T., Zhang D., Cai M., Wang C., Wu Z., Ying Z., Wu J., Li M., Xie D., Li J. (2015). Golgi phosphoprotein 3 (GOLPH3) promotes hepatocellular carcinoma cell aggressiveness by activating the NF-kappaB pathway. J. Pathol..

[134] Lee T.H., Shank J., Cusson N., Kelliher M.A. (2004). The kinase activity of Rip1 is not required for tumor necrosis factor-alpha-induced IkappaB kinase or p38 MAP kinase activation or for the ubiquitination of Rip1 by Traf2. J. Biol. Chem..

[135] Knop J., Martin M.U. (1999). Effects of IL-1 receptor-associated kinase (IRAK) expression on IL-1 signaling are independent of its kinase activity. FEBS Lett..

[136] Shim J.H., Xiao C., Paschal A.E., Bailey S.T., Rao P., Hayden M.S., Lee K.Y., Bussey C., Steckel M., Tanaka N. (2005). TAK1, but not TAB1 or TAB2, plays an essential role in multiple signaling pathways in vivo. Genes Dev..

[137] Xia Z.P., Sun L., Chen X., Pineda G., Jiang X., Adhikari A., Zeng W., Chen Z.J. (2009). Direct activation of protein kinases by unanchored polyubiquitin chains. Nature.

[138] Skaug B., Chen J., Du F., He J., Ma A., Chen Z.J. (2011). Direct, noncatalytic mechanism of IKK inhibition by A20. Mol. Cell.

[139] Komander D., Reyes-Turcu F., Licchesi J.D., Odenwaelder P., Wilkinson K.D., Barford D. (2009). Molecular discrimination of structurally equivalent Lys 63-linked and linear polyubiquitin chains. EMBO Rep..

[140] Kulathu Y., Akutsu M., Bremm A., Hofmann K., Komander D. (2009). Two-sided ubiquitin binding explains specificity of the TAB2 NZF domain. Nat. Struct. Mol. Biol..

[141] Sato Y., Yoshikawa A., Yamashita M., Yamagata A., Fukai S. (2009). Structural basis for specific recognition of Lys 63-linked polyubiquitin chains by NZF domains of TAB2 and TAB3. EMBO J..

[142] Ori D., Kato H., Sanjo H., Tartey S., Mino T., Akira S., Takeuchi O. (2013). Essential roles of K63-linked polyubiquitin-binding proteins TAB2 and TAB3 in B cell activation via MAPKs. J. Immunol..

[143] Wan Y.Y., Chi H., Xie M., Schneider M.D., Flavell R.A. (2006). The kinase TAK1 integrates antigen and cytokine receptor signaling for T cell development, survival and function. Nat. Immunol..

[144] Sato S., Sanjo H., Takeda K., Ninomiya-Tsuji J., Yamamoto M., Kawai T., Matsumoto K., Takeuchi O., Akira S. (2005). Essential function for the kinase TAK1 in innate and adaptive immune responses. Nat. Immunol..

[145] Schrofelbauer B., Polley S., Behar M., Ghosh G., Hoffmann A. (2012). NEMO ensures signaling specificity of the pleiotropic IKKbeta by directing its kinase activity toward IkappaBalpha. Mol. Cell.

[146] Wu Z., Berlemann L.A., Bader V., Sehr D.A., Dawin E., Covallero A., Meschede J., Angersbach L., Showkat C., Michaelis J.B. (2022). LUBAC assembles a ubiquitin signaling platform at mitochondria for signal amplification and transport of NF-kappaB to the nucleus. EMBO J..

[147] Hua F., Hao W., Wang L., Song K., Hasan A., Wu Y., Li K., Lin Z., Sun Y., Li S. (2024). Linear ubiquitination mediates coronavirus NSP14-induced NF-kappaB activation. Cell Commun. Signal.

[148] Yang J., Lin Y., Guo Z., Cheng J., Huang J., Deng L., Liao W., Chen Z., Liu Z., Su B. (2001). The essential role of MEKK3 in TNF-induced NF-kappaB activation. Nat. Immunol..

[149] Lee F.S., Hagler J., Chen Z.J., Maniatis T. (1997). Activation of the IkappaB alpha kinase complex by MEKK1, a kinase of the JNK pathway. Cell.

[150] Prajapati S., Gaynor R.B. (2002). Regulation of Ikappa B kinase (IKK)gamma /NEMO function by IKKbeta -mediated phosphorylation. J. Biol. Chem..

[151] Huang T.T., Wuerzberger-Davis S.M., Wu Z.H., Miyamoto S. (2003). Sequential modification of NEMO/IKKgamma by SUMO-1 and ubiquitin mediates NF-kappaB activation by genotoxic stress. Cell.

[152] Sebban H., Yamaoka S., Courtois G. (2006). Posttranslational modifications of NEMO and its partners in NF-kappaB signaling. Trends Cell Biol..

[153] Yoshikawa A., Sato Y., Yamashita M., Mimura H., Yamagata A., Fukai S. (2009). Crystal structure of the NEMO ubiquitin-binding domain in complex with Lys 63-linked di-ubiquitin. FEBS Lett..

[154] Catici D.A., Horne J.E., Cooper G.E., Pudney C.R. (2015). Polyubiquitin Drives the Molecular Interactions of the NF-kappaB Essential Modulator (NEMO) by Allosteric Regulation. J. Biol. Chem..

[155] Catici D.A., Amos H.E., Yang Y., van den Elsen J.M., Pudney C.R. (2016). The red edge excitation shift phenomenon can be used to unmask protein structural ensembles: Implications for NEMO-ubiquitin interactions. FEBS J..

[156] Hauenstein A.V., Xu G., Kabaleeswaran V., Wu H. (2017). Evidence for M1-Linked Polyubiquitin-Mediated Conformational Change in NEMO. J. Mol. Biol..

[157] Ko M.S., Cohen S.N., Polley S., Mahata S.K., Biswas T., Huxford T., Ghosh G. (2022). Regulatory subunit NEMO promotes polyubiquitin-dependent induction of NF-kappaB through a targetable second interaction with upstream activator IKK2. J. Biol. Chem..

[158] Michel M.A., Scutts S., Komander D. (2024). Secondary interactions in ubiquitin-binding domains achieve linkage or substrate specificity. Cell Rep..

[159] Scholefield J., Henriques R., Savulescu A.F., Fontan E., Boucharlat A., Laplantine E., Smahi A., Israel A., Agou F., Mhlanga M.M. (2016). Super-resolution microscopy reveals a preformed NEMO lattice structure that is collapsed in incontinentia pigmenti. Nat. Commun..

[160] Shaffer R., DeMaria A.M., Kagermazova L., Liu Y., Babaei M., Caban-Penix S., Cervantes A., Jehle S., Makowski L., Gilmore T.D. (2019). A Central Region of NF-kappaB Essential Modulator Is Required for IKKbeta-Induced Conformational Change and for Signal Propagation. Biochemistry.

[161] Chen Z.J. (2012). Ubiquitination in signaling to and activation of IKK. Immunol. Rev..

[162] Goel S., Oliva R., Jeganathan S., Bader V., Krause L.J., Kriegler S., Stender I.D., Christine C.W., Nakamura K., Hoffmann J.E. (2023). Linear ubiquitination induces NEMO phase separation to activate NF-kappaB signaling. Life Sci. Alliance.

[163] DiRusso C.J., DeMaria A.M., Wong J., Wang W., Jordanides J.J., Whitty A., Allen K.N., Gilmore T.D. (2023). A conserved core region of the scaffold NEMO is essential for signal-induced conformational change and liquid-liquid phase separation. J. Biol. Chem..

[164] Bracken C.P., Whitelaw M.L., Peet D.J. (2005). Activity of hypoxia-inducible factor 2alpha is regulated by association with the NF-kappaB essential modulator. J. Biol. Chem..

[165] Nowicka A.M., Hauselmann I., Borsig L., Bolduan S., Schindler M., Schraml P., Heikenwalder M., Moch H. (2016). A novel pVHL-independent but NEMO-driven pathway in renal cancer promotes HIF stabilization. Oncogene.

[166] Kondylis V., Polykratis A., Ehlken H., Ochoa-Callejero L., Straub B.K., Krishna-Subramanian S., Van T.M., Curth H.M., Heise N., Weih F. (2015). NEMO Prevents Steatohepatitis and Hepatocellular Carcinoma by Inhibiting RIPK1 Kinase Activity-Mediated Hepatocyte Apoptosis. Cancer Cell.

[167] Vlantis K., Wullaert A., Polykratis A., Kondylis V., Dannappel M., Schwarzer R., Welz P., Corona T., Walczak H., Weih F. (2016). NEMO Prevents RIP Kinase 1-Mediated Epithelial Cell Death and Chronic Intestinal Inflammation by NF-kappaB-Dependent and -Independent Functions. Immunity.

[168] Brahler S., Ising C., Barrera Aranda B., Hohne M., Schermer B., Benzing T., Brinkkoetter P.T. (2015). The NF-kappaB essential modulator (NEMO) controls podocyte cytoskeletal dynamics independently of NF-kappaB. Am. J. Physiol.-Ren. Physiol..

[169] Fusco F., Pescatore A., Conte M.I., Mirabelli P., Paciolla M., Esposito E., Lioi M.B., Ursini M.V. (2015). EDA-ID and IP, two faces of the same coin: How the same IKBKG/NEMO mutation affecting the NF-kappaB pathway can cause immunodeficiency and/or inflammation. Int. Rev. Immunol..

[170] Hubeau M., Ngadjeua F., Puel A., Israel L., Feinberg J., Chrabieh M., Belani K., Bodemer C., Fabre I., Plebani A. (2011). New mechanism of X-linked anhidrotic ectodermal dysplasia with immunodeficiency: Impairment of ubiquitin binding despite normal folding of NEMO protein. Blood.

[171] Rahighi S., Iyer M., Oveisi H., Nasser S., Duong V. (2022). Structural basis for the simultaneous recognition of NEMO and acceptor ubiquitin by the HOIP NZF1 domain. Sci. Rep..

[172] Bal E., Laplantine E., Hamel Y., Dubosclard V., Boisson B., Pescatore A., Picard C., Hadj-Rabia S., Royer G., Steffann J. (2017). Lack of interaction between NEMO and SHARPIN impairs linear ubiquitination and NF-kappaB activation and leads to incontinentia pigmenti. J. Allergy Clin. Immunol..

[173] Hsu A.P., Zerbe C.S., Foruraghi L., Iovine N.M., Leiding J.W., Mushatt D.M., Wild L., Kuhns D.B., Holland S.M. (2018). IKBKG (NEMO) 5’ Untranslated Splice Mutations Lead to Severe, Chronic Disseminated Mycobacterial Infections. Clin. Infect. Dis..

[174] Shariq M., Quadir N., Alam A., Zarin S., Sheikh J.A., Sharma N., Samal J., Ahmad U., Kumari I., Hasnain S.E. (2023). The exploitation of host autophagy and ubiquitin machinery by Mycobacterium tuberculosis in shaping immune responses and host defense during infection. Autophagy.

[175] Slowicka K., Vereecke L., van Loo G. (2016). Cellular Functions of Optineurin in Health and Disease. Trends Immunol..

[176] Nakazawa S., Oikawa D., Ishii R., Ayaki T., Takahashi H., Takeda H., Ishitani R., Kamei K., Takeyoshi I., Kawakami H. (2016). Linear ubiquitination is involved in the pathogenesis of optineurin-associated amyotrophic lateral sclerosis. Nat. Commun..

[177] Marin-Rubio J.L., Raote I., Inns J., Dobson-Stone C., Rajan N. (2023). CYLD in health and disease. Dis. Model. Mech..

[178] Verboom L., Hoste E., van Loo G. (2021). OTULIN in NF-kappaB signaling, cell death, and disease. Trends Immunol..

[179] Karri U., Harasimowicz M., Carpio Tumba M., Schwartz D.M. (2024). The Complexity of Being A20: From Biological Functions to Genetic Associations. J. Clin. Immunol..

[180] Castanier C., Arnoult D. (2011). Mitochondrial localization of viral proteins as a means to subvert host defense. Biochim. Biophys. Acta.

[181] Wang Y., Cui L., Yang G., Zhan J., Guo L., Chen Y., Fan C., Liu D., Guo D. (2019). Hepatitis B e Antigen Inhibits NF-kappaB Activity by Interrupting K63-Linked Ubiquitination of NEMO. J. Virol..

[182] Chen Y., He L., Peng Y., Shi X., Chen J., Zhong J., Chen X., Cheng G., Deng H. (2015). The hepatitis C virus protein NS3 suppresses TNF-alpha-stimulated activation of NF-kappaB by targeting LUBAC. Sci. Signal..

[183] Muscolino E., Schmitz R., Loroch S., Caragliano E., Schneider C., Rizzato M., Kim Y.H., Krause E., Juranic Lisnic V., Sickmann A. (2020). Herpesviruses induce aggregation and selective autophagy of host signalling proteins NEMO and RIPK1 as an immune-evasion mechanism. Nat. Microbiol..

[184] Brady G., Haas D.A., Farrell P.J., Pichlmair A., Bowie A.G. (2017). Molluscum Contagiosum Virus Protein MC005 Inhibits NF-kappaB Activation by Targeting NEMO-Regulated IkappaB Kinase Activation. J. Virol..

[185] Biswas S., Shisler J.L. (2017). Molluscum Contagiosum Virus MC159 Abrogates cIAP1-NEMO Interactions and Inhibits NEMO Polyubiquitination. J. Virol..

[186] Chen J., Wang D., Sun Z., Gao L., Zhu X., Guo J., Xu S., Fang L., Li K., Xiao S. (2019). Arterivirus nsp4 Antagonizes Interferon Beta Production by Proteolytically Cleaving NEMO at Multiple Sites. J. Virol..

[187] Chen S., Tian J., Li Z., Kang H., Zhang J., Huang J., Yin H., Hu X., Qu L. (2019). Feline Infectious Peritonitis Virus Nsp5 Inhibits Type I Interferon Production by Cleaving NEMO at Multiple Sites. Viruses.

[188] Wenzel J., Lampe J., Muller-Fielitz H., Schuster R., Zille M., Muller K., Krohn M., Korbelin J., Zhang L., Ozorhan U. (2021). The SARS-CoV-2 main protease M(pro) causes microvascular brain pathology by cleaving NEMO in brain endothelial cells. Nat. Neurosci..

[189] Hameedi M.A., Prates E.T., Garvin M.R., Mathews I.I., Amos B.K., Demerdash O., Bechthold M., Iyer M., Rahighi S., Kneller D.W. (2022). Structural and functional characterization of NEMO cleavage by SARS-CoV-2 3CLpro. Nat. Commun..

[190] Wu J., Shi Y., Pan X., Wu S., Hou R., Zhang Y., Zhong T., Tang H., Du W., Wang L. (2021). SARS-CoV-2 ORF9b inhibits RIG-I-MAVS antiviral signaling by interrupting K63-linked ubiquitination of NEMO. Cell Rep..

[191] Nishitsuji H., Iwahori S., Ohmori M., Shimotohno K., Murata T. (2022). Ubiquitination of SARS-CoV-2 NSP6 and ORF7a Facilitates NF-kappaB Activation. mBio.

[192] Song J., Guo Y., Wang D., Quan R., Wang J., Liu J. (2024). Seneca Valley virus 3C protease cleaves OPTN (optineurin) to Impair selective autophagy and type I interferon signaling. Autophagy.

[193] Sun D., Wu R., Zheng J., Li P., Yu L. (2018). Polyubiquitin chain-induced p62 phase separation drives autophagic cargo segregation. Cell Res..

[194] Kageyama S., Gudmundsson S.R., Sou Y.S., Ichimura Y., Tamura N., Kazuno S., Ueno T., Miura Y., Noshiro D., Abe M. (2021). p62/SQSTM1-droplet serves as a platform for autophagosome formation and anti-oxidative stress response. Nat. Commun..

[195] Chiaravalli J., Fontan E., Fsihi H., Coic Y.M., Baleux F., Veron M., Agou F. (2011). Direct inhibition of NF-kappaB activation by peptide targeting the NOA ubiquitin binding domain of NEMO. Biochem. Pharmacol..

[196] Vincendeau M., Hadian K., Messias A.C., Brenke J.K., Halander J., Griesbach R., Greczmiel U., Bertossi A., Stehle R., Nagel D. (2016). Inhibition of Canonical NF-kappaB Signaling by a Small Molecule Targeting NEMO-Ubiquitin Interaction. Sci. Rep..

